# Synthesis and Evaluation of New 1,3,4-Thiadiazole Derivatives as Potent Antifungal Agents

**DOI:** 10.3390/molecules23123129

**Published:** 2018-11-29

**Authors:** Ahmet Çağrı Karaburun, Ulviye Acar Çevik, Derya Osmaniye, Begüm Nurpelin Sağlık, Betül Kaya Çavuşoğlu, Serkan Levent, Yusuf Özkay, Ali Savaş Koparal, Mustafa Behçet, Zafer Asım Kaplancıklı

**Affiliations:** 1 Department of Pharmaceutical Chemistry, Faculty of Pharmacy, Anadolu University, Eskişehir 26470, Turkey; ackarabu@anadolu.edu.tr (A.C.K.); dosmaniye@anadolu.edu.tr (D.O.); bnsaglik@anadolu.edu.tr (B.N.S.); betulkaya@anadolu.edu.tr (B.K.Ç.); serkanlevent@anadolu.edu.tr (S.L.); yozkay@anadolu.edu.tr (Y.Ö.); zakaplan@anadolu.edu.tr (Z.A.K.); 2 Doping and Narcotic Compounds Analysis Laboratory, Faculty of Pharmacy, Anadolu University, Eskişehir 26470, Turkey; 3 Open Education Faculty, Anadolu University, Eskişehir 26470, Turkey; askopara@anadolu.edu.tr; 4 Department of Medical Microbiology, Faculty of Medicine, Abant İzzet Baysal University, Bolu 14280, Turkey; mustafabehcet@ibu.edu.tr

**Keywords:** 1,3,4-thiadiazole, thione-thiol tautomerism, docking, antifungal, 14-α-sterol demethylase, ADME

## Abstract

With the goal of obtaining a novel bioactive compound with significant antifungal activity, a series of 1,3,4-thiadiazole derivatives (**3a**–**3l**) were synthesized and characterized. Due to thione-thiol tautomerism in the intermediate compound **2**, type of substitution reaction in the final step was determined by two-dimensional (2D) NMR. In vitro antifungal activity of the synthesized compounds was evaluated against eight *Candida* species. The active compounds **3k** and **3l** displayed very notable antifungal effects. The probable mechanisms of action of active compounds were investigated using an ergosterol quantification assay. Docking studies on 14-α-sterol demethylase enzyme were also performed to investigate the inhibition potency of compounds on ergosterol biosynthesis. Theoretical absorption, distribution, metabolism, and excretion (ADME) predictions were calculated to seek their drug likeness of final compounds. The results of the antifungal activity test, ergosterol biosynthesis assay, docking study, and ADME predictions indicated that the synthesized compounds are potential antifungal agents, which inhibit ergosterol biosynthesis probably interacting with the fungal 14-α-sterol demethylase.

## 1. Introduction

Invasive fungal infections due to *Candida* species are being increasingly shown as a main reason of morbidity and mortality in the healthcare environment [1,2]. These infections typically occur due to the effect of five most general pathogens *C. glabrata*, *C. albicans*, *C. krusei*, *C. parapsilosis*, and *C. tropicalis*. Each of these organisms has unique virulence potency, antifungal susceptibility and epidemiology; however, when considered as a whole, important organism-related infections are often referred to as invasive candidiasis. The high prevalence of antifungal resistance of pathogenic *Candida* species to existing drugs reduces the effectiveness of treatment; thus, the development of alternative antifungal agents is required [3,4]. 

Due to the low incidence of fungal infections, the rate of development of new antifungal agents is lower than that of antibacterial agents. In addition, fungi are eukaryotic (as are their human hosts) and, thus, it is difficult to investigate antifungal targets. However, detailed information on the nature of fungal cells contributed to the understanding of the mechanisms of action of many antifungal agents in addition to a variety of fungal infections [5,6]. Efforts are expected to reduce toxicity, increase bioavailability, improve the antifungal spectrum and combat resistance, and enhance the efficacy of existing antifungals. In fact, elucidation of the mechanism of action of a potential antifungal compound can reduce the time period from lead compound to drug candidate agent [7]. 

Currently, the available antifungal agents to treat *Candida* infections can be divided into four categories based on their mode of action, including azoles, polyenes, echinocandins, and antimetabolites [8,9]. Among these agents, azole groups are most widely used in antifungal therapy [10].

The 1,3,4-thiadiazole compound is a five-membered heterocyclic scaffold including diverse physicochemical properties. It is a mesoionic system associated with the discrete regions of positive and negative charges leading to σ and π electrons and highly polarizable derivatives [11]. This distinguishing feature allows mesoionic compounds to effectively cross cellular membranes and interact with biological molecules in unique ways, which highlights the high potential of this ring system in medicinal chemistry [12]. Furthermore, it was reported that the toxophoric –N–C–S moiety of the 1,3,4-thiadiazoles is probably responsible for a broad range of biological activity [13]. For instance, 1,3,4-thiadiazole derivatives were extensively used as pesticides with a wide range of biocidal properties, among which antifungal activities attracted particular attention as part of a comprehensive project for the development of agricultural fungicides [14,15,16,17,18]. The good liposolubility of the sulfur atom in the thiadiazole compounds might also have a positive effect on the biological activity [19,20,21].

**Figure 1:**
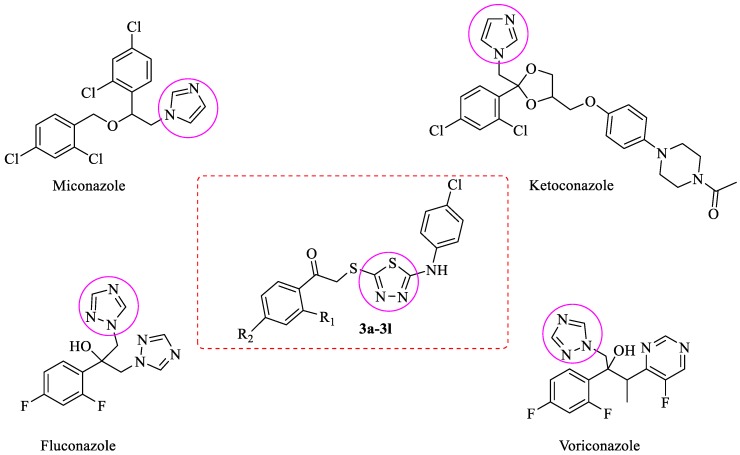
Chemical structures of targeted compounds and some antifungal drugs which are in clinical use.

Thiadiazole is a bioisoster of triazole and imidazole, which are members of the azole group with antifungal importance (Figure 1). Imidazole- and triazole-based synthetic antifungal drugs prevent the conversion of lanosterol to ergosterol and inhibit the enzyme cytochrome P450 14-α-demethylase enzyme [13,22,23,24,25,26,27]. Based on the bioisosterism and antifungal potency of azole-type compounds, researchers reported several studies including thiadiazole derivatives and their antifungal activity [28,29,30]. From this point of view, in our previous paper, we reported the synthesis and antifungal activity evaluation of some oxadiazole–thiadiazole derivatives, which target the P450 14-α-demethylase enzyme [28]. Results of our study encouraged us to synthesize more compounds including thiadiazole nuclei. Thus, we prepared a new series of thiadiazole derivatives, and tested their antifungal activities with in vitro and in silico studies.

## 2. Results and Discussion

### 2.1. Chemistry

**Scheme 1:**
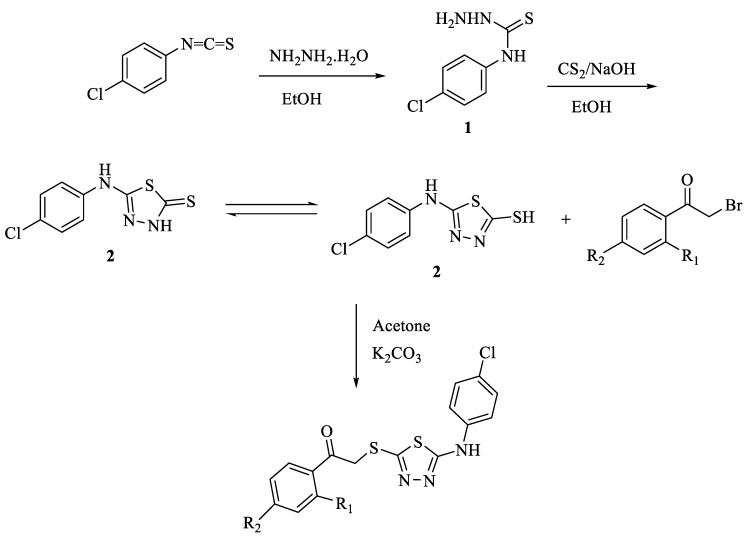
The synthetic route of the compounds (**3a**–**3l**).

The target compounds (**3a**–**3l**) were prepared via three steps, starting from 1-chloro-4-isothiocyanatobenzene. In the first step, in an ice bath, 4-chlorophenyl isothiocyanate reacted with hydrazine hydrate to give *N*-(4-chlorophenyl)hydrazinecarbothioamide (**1**). Then, compound **1** reacted with CS_2_ under alkaline conditions, such as NaOH. The synthesized compound **2** (5-((4-chlorophenyl)amino)-1,3,4-thiadiazole-2-thiol) was then converted to the target compounds **3a**–**3l** upon reacting with 2-bromoacetophenone derivatives in acetone. The synthetic method employed to synthesize these compounds is given in Scheme 1.

Compounds **3a**–**3l** were confirmed by infrared (IR), ^1^H-NMR, ^13^C-NMR, and LC–MS (see Supplementary Materials). The IR spectra of compounds **3a**–**3l** showed N–H absorption bands in the range of 3244 to 3265 cm^−1^. The stretching bands for C=O were observed between 1637 and 1689 cm^−1^ as expected. The stretching bands for 1,4-disubstituted benzene were determined at 810–852 cm^−1^.

For the ^1^H-NMR spectra of compounds **3a**–**3l**, the signal of the N–H proton was seen in the region of 10.51–10.55 ppm as a singlet. The signal of S–CH_2_ protons was observed at 4.69–5.01 ppm as a singlet peak. The hydrogens of two phenyl rings and other aliphatic hydrogens were observed in the expected areas.

The ^13^C-NMR spectra exhibited different types of signals at various ppm which were assigned for the different types of carbons of all the target compounds. All compounds (**3a**–**3l**) gave rise to the molecular-ion peak [M + H]^+^ in the LC–MS-8040 tandem mass system, which confirmed the calculated mass data. 

**Figure 2:**
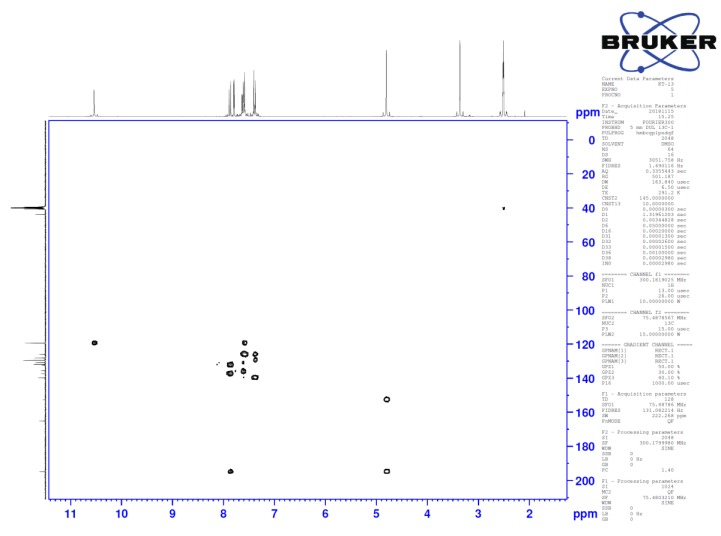
HMBC plot of compound **3l**.

In addition to structure elucidations using routine spectroscopic methods, two-dimensional (2D) NMR heteronuclear multiple bond correlation (HMBC) analysis was also performed for a selected final compound (**3l**). As seen in Scheme 1, compound **2** has two tautomeric forms. Thus, it can be suspected whether the substitution reaction in the final step is N-substitution or S-substitution. In fact it is known that –SH proton is more acidic than –NH proton and, therefore, the –SH group is more susceptible to nucleophilic substitution than the –NH group in alkaline conditions. Nevertheless, we carried out the 2D NMR analysis of compound **3l** to determine whether the substitution pathway was a sulfur atom or a nitrogen atom. At first glance, in the ^13^ C-NMR spectrum of compound **3l**, 165-ppm and 194-ppm peaks may be considered as belonging to carbonyl and thione groups, respectively. In this case, the presence of the thione group indicates that the reaction occurred as N-alkylation. However, in the HMBC plot (Figure 2) there is an interaction between the carbon peak at 194 ppm and the hydrogen peak at 7.9 ppm, belonging to the sixth position of 2,4-dichlorobenzene. Furthermore, it is clearly observed that hydrogens of methylene (–CH_2_) at 4.8 ppm correlate with the carbons at 194 ppm and 152 ppm, and do not show any interaction with the carbon at 165 ppm. Therefore, it can easily be suggested that the peak at 194 ppm belongs to the carbonyl group instead of the thione group, and the peaks at 152 ppm and 165 ppm belong to carbons at the second and fifth position of the thiadiazole ring. As a result, HMBC analysis reveals that the reaction in the final step occurs as an S-substitution as expected.

### 2.2. Biological Activity Screening

#### 2.2.1. Antifungal Activity

The in vitro antifungal activity results of the title compounds against *C. albicans* (ATCC 90028), *C. crusei* (ATCC 6258), *C. parapsilosis* (ATCC 22019), *C. tropicalis* (ATCC 13803), *C. albicans* (ATCC 10231), *C*. *glabrata* (ATCC 2001), *C. famata* (Abant İzzet Baysal University Medical Faculty Hospital, clinical isolate), and *C. lusitaniae* (Abant İzzet Baysal University Medical Faculty Hospital, clinical isolate) are listed in Table 1, where fluconazole was used as a control.

Compounds **3e**, **3i**, and **3j** were the least active among this series as antifungal agents against all tested fungus strains. Compound **3a** exhibited same activity when compared with the standard drug fluconazole against four tested fungus strains. Compounds **3f** and **3g** displayed very similar antifungal activity against the same *Candida* strains. The compounds **3b**, **3c**, **3d**, and **3h** also exhibited similar anticandidal activity to fluconazole against most of the tested fungus strains.

Compound **3l** bearing 2,4-dichlorophenyl was the most active compound in the series against *C. albicans* ATCC 10231 with a minimum inhibitory concentration (MIC) value of 5 µg/mL. Moreover, compound **3l** had significant antifungal activity against *C. crusei* ATCC 6258, *C. glabrata* ATCC 2001, and *C. famata* with an MIC value of 10 µg/mL. Compound **3k** bearing 2,4-difluorophenyl also possessed good activity against *C. albicans* ATCC 10231, *C. crusei* ATCC 6528, and *C. famata* with an MIC value of 10 µg/mL. Compounds **3k** and **3l** also exhibited comparable antifungal activity to the standard drug fluconazole against the other *Candida* strains. However, compounds **3f** and **3g** with chloro and fluoro substituents at the fourth position of the phenyl ring showed lower activity than compounds **3k** and **3l**. These findings indicated that the antifungal activity of the compounds is enhanced when halogens such as chloro or fluoro are added to the second position of the phenyl ring.

#### 2.2.2. Quantification of Ergosterol Level

Ergosterol, a major sterol of the fungal cell membrane, is essential for membrane integrity and fluidity, nutrient transport, and chitin synthesis. Furthermore, trace amounts of ergosterol are required as a “sparking” function to allow progression through the cell cycle. The difference in composition of sterols between mammalian cells, which primarily feature cholesterol, and fungal cells, primarily ergosterol, is what allows for many of the popular antifungal treatments in use today to be effective [31,32].

The present study was undertaken to investigate the probable mechanism of action of the antifungal activity of newly synthesized thiadiazole compounds **3k** and **3l**. It is known that current therapies of fungal infections mostly target the ergosterol biosynthesis pathway. The reason for this medicinal strategy is the significance of ergosterol, which causes end-of-life functions as a result of the inhibition of its biosynthesis. 

For quantitative determination of the ergosterol level of *C. albicans* ATCC 10231, we used an LC–MS/MS method. Total intracellular sterols were extracted as reported by Breivik and Owades [33]. Ergosterol standard (Product No: 45480, Sigma-Aldrich, Steinheim, Germany) was used to quantify the ergosterol level in both inhibitor-free (negative control) and inhibitor-including samples. The most active compounds **3k** and **3l** and the reference drug were tested in the concentration range of 2.5 µg/mL to 40 µg/mL. The level of ergosterol in the negative control was considered as 100%. Both compounds were analyzed four times for all concentrations. Then, the results were presented as means ± standard deviation (SD) (Table 2). 

The studies to determine ergosterol level indicated that compounds **3k** and **3l** and the reference agent possess an important effect on ergosterol level. Thus, the results show that the level of ergestrol decreases with increasing compound concentration. As a result, it can be obviously suggested that compounds **3k** and **3l** may have a role in the biosynthesis of ergosterol.

#### 2.2.3. Molecular Docking Studies

Compounds **3k** and **3l** were found as the most potent antifungal derivatives in the series, and an in vitro ergosterol quantification assay was carried out for them. In order to evaluate the in silico features of these compounds, docking studies were performed on the 14-α-sterol demethylase enzyme, which is a key enzyme for ergosterol biosynthesis in fungi.

It can be understood by looking at the anticandidal activity results that compounds **3k** and **3l** were the most active derivatives, especially against *C. albicans*, with MIC_50_ values of 5 and 10 µg/mL. Therefore, in silico docking studies were carried out using the X-ray crystal structure originating from *C. albicans* (Protein Data Bank identifier (PDB ID): 5FSA) [34], which was obtained from the Protein Data Bank server (www.pdb.org).

**Figure 3:**
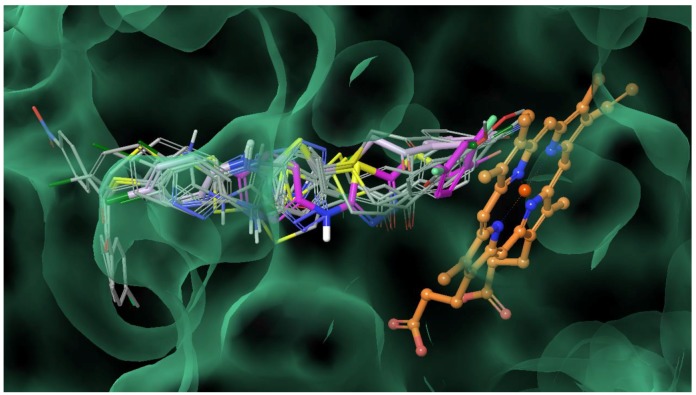
The superimposition of all compounds (**3a**–**3l**) in the active region of 14-α-sterol demethylase (Protein Data Bank (PDB) code: 5FSA). The heme molecule is colored orange, while compounds **3k** and **3l** are colored purple and pink, respectively.

Figure 3 presents the superimposition of all compounds in the active region of 14-α-sterol demethylase. It was determined that compounds **3k** and **3l** settle down in the active region in different conformations, which may be the reason for more interactions than other compounds.

**Figure 4:**
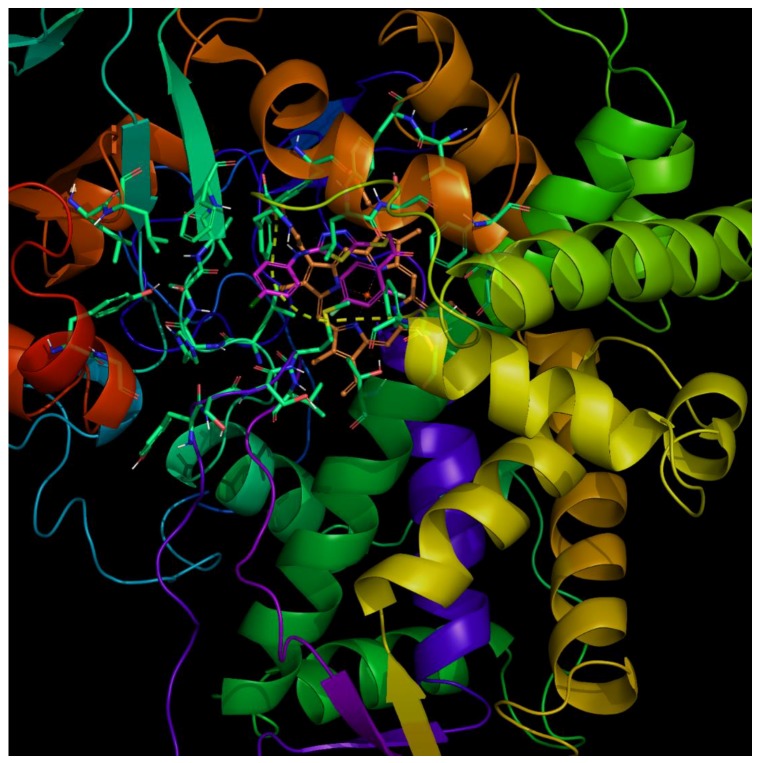
Active compound **3k** in the active region of 14-α-sterol demethylase (PDB code: 5FSA). The inhibitor is colored purple and the heme molecule is colored orange.

**Figure 5:**
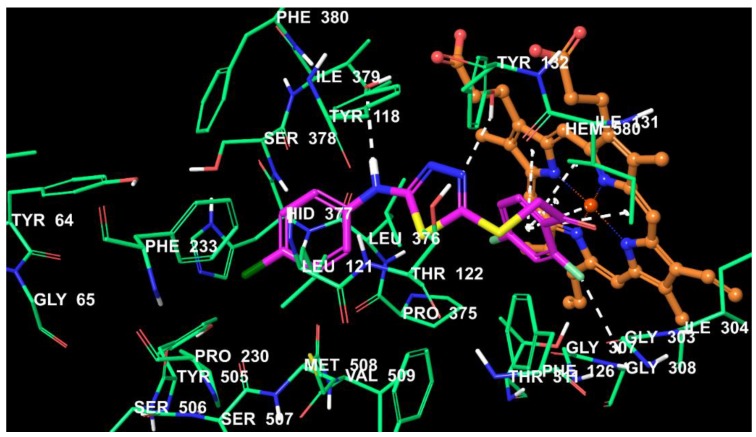
The interacting mode of compound **3k** in the active region of 14-α-sterol demethylase (PDB code: 5FSA). The inhibitor is colored purple, and the heme molecule is colored orange.

**Figure 6:**
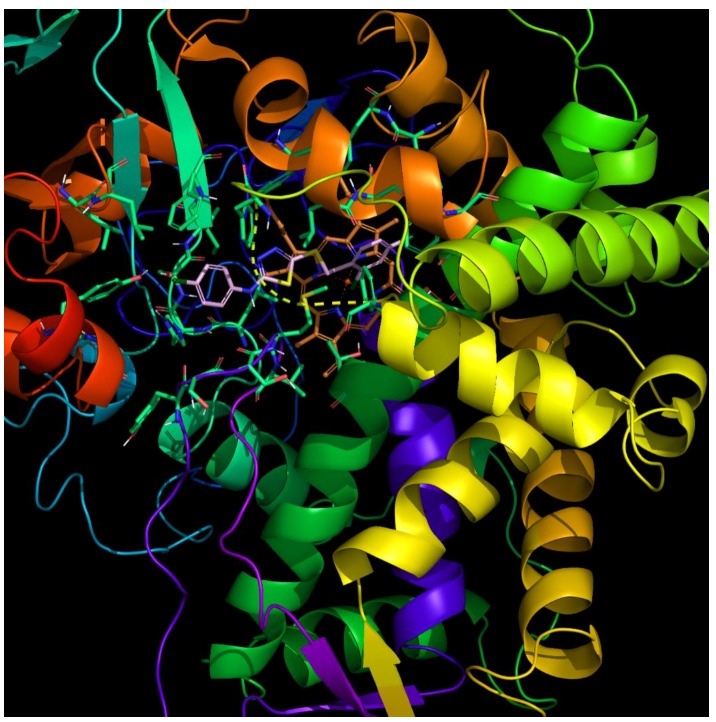
Active compound **3l** in the active region of 14-α-sterol demethylase (PDB code: 5FSA). The inhibitor is colored pink and the heme molecule is colored orange.

**Figure 7:**
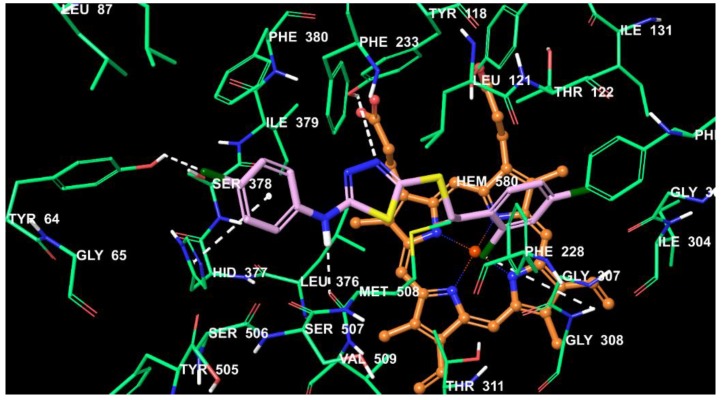
The interacting mode of compound **3l** in the active region of 14-α-sterol demethylase (PDB code: 5FSA). The inhibitor is colored pink and the heme molecule is colored orange.

The docking poses of compounds **3k** and **3l** are presented in Figure 4, Figure 5, Figure 6 and Figure 7. It can be seen that there are two common interactions for these compounds. The first of them is related to the thiadiazole ring. The nitrogen atom at the third position of the thiadiazole ring forms a hydrogen bond with the hydroxyl group of Tyr132 for compound **3k**, whereas this hydrogen bond is observed with the hydroxyl group of Tyr118 for compound **3l**. The other common interaction is created between the amino group in the structure and the hydroxyl group of Tyr118 for compound **3k**. This interaction is observed with the carbonyl of Met508 in compound **3l**. 

The main structural difference of these compounds is the presence of halogen atoms at the C2 and C4 positions of the phenyl ring. It can be seen that this disubstituted phenyl ring is in a π–π interaction with the heme molecule. While compound **3k** carries fluorine atoms at the C2 and C4 positions, compound **3l** has chlorine atoms. It can be suggested based on anticandidal activity and molecular docking studies that the presence of halogen atoms, especially at the C2 position of the phenyl ring, strengthens the antifungal activity and ergosterol biosynthesis. As can be seen from the docking poses (Figure 5 and Figure 7), the ortho fluorine and chlorine atoms establish a halogen bond with the hydrogen of the amino group of Gly308 in compounds **3k** and **3l**, respectively. 

Furthermore, by looking at Figure 7, it can be seen that compound **3l** has two additional interactions compared to compound **3k**. These additional two interactions are formed by the *para*-chlorophenyl ring next to the amino group in the structure. The *para*-chlorophenyl ring is in interaction with the imidazole ring of Hid377 via a π–π interaction. The other interaction is related to the chlorine atom at the C4 position of the phenyl ring. The chlorine atom creates a halogen bond with the hydrogen of the hydroxyl group of Tyr64. It was theorized that compound **3l** can show these additional interactions compared to compound **3k** owing to its different conformation caused by the volume of chlorine atoms at the C2 and C4 positions of the phenyl ring.

#### 2.2.4. Theoretical Determination of ADME Properties

ADME refers to the absorption, distribution, metabolism, and excretion of a molecule in an organism. All these characteristics are very important for any drug. Having favorable ADME characteristics is one of the most daunting hurdles for drug development. Thus, early optimization is very essential in this process. A waste of time and money in the later stages of drug development can be prevented thanks to early optimization. The identification and elimination of unfavorable compounds makes the research process more cost-effective and efficient [35]. For this reason, the prediction of the pharmacokinetic properties of new drug candidates as early as possible in the drug development process is very important. 

ADME parameters of synthesized compounds (**3a**–**3l**) were calculated with the help of *QikProp 4.8* software [36]. *QikProp* also allows providing acceptable ranges for comparing the predicted properties of compounds with those of 95% of known drugs, allowing the estimation of drug-likeness properties. The drug-likeness of a compounds was assessed according to Lipinski’s Rule of Five [37], which considers molecular weight (<500 Da), number of hydrogen-bond acceptors (≤10) and donors (≤5), and octanol/water partition coefficient (≤5), and Jorgensen’s rule of three [38], which regards logS (>−5.7), PCaco (>22 nm/s), and primary metabolites (PM) (<7). The violations of these rules are essential for the optimization of biologically active compounds and should not be more than 1. 

Table 3 presents predicted ADME properties of all compounds, and this table contains the following parameters: molecular weight (MW), number of rotatable bonds (RB), dipole moment (DM), molecular volume (MV), number of hydrogen donors (DHB), number of hydrogen acceptors (AHB), polar surface area (PSA), octanol/water partition coefficient (log P), aqueous solubility (log S), apparent Caco-2 cell permeability (PCaco), number of likely primer metabolic reactions (PM), percentage of human oral absorption (%HOA), and the violations of rules of three (VRT) and five (VRF).

The theoretical calculations of ADME parameters (molecular weight, log P, topological polar surface area (tPSA), number of hydrogen donors (nON), number of hydrogen acceptors (nOHNH), and molecular volume (MV)) are presented in Table 3 along with the violations of Lipinski’s and Jorgensen’s rules. According to these data, all compounds (**3a**–**3l**) follow both rules, causing no more than one violation. Thus, it can be suggested that the synthesized compounds may possess a good pharmacokinetic profile, increasing their pharmacological importance. 

Consequently, according to predictions of the ADME properties, it can be suggested that the final compounds may have a good pharmacokinetic profile.

## 3. Materials and Methods 

### 3.1. Chemistry

All chemicals used in the synthesis studies were obtained from Merck Chemicals (Merck KGaA, Darmstadt, Germany) or Sigma-Aldrich Chemicals (Sigma-Aldrich Corp., St. Louis, Mo., USA). The MP90 digital melting point apparatus (Mettler Toledo, OH, USA) was used to determine the melting points (MPs) of the resulting compounds, which were presented uncorrected. Bruker 300-MHz and 75-MHz digital Fourier transform (FT) NMR spectrometers (Bruker Bioscience, Billerica, MA, USA) in dimethyl sulfoxide (DMSO-*d_6_*) were used to record the ^1^H NMR and ^13^C NMR spectra, respectively. In the NMR spectra, splitting patterns were determined as follows: s, singlet; d, doublet; t, triplet; dd, double doublet; m, multiplet. Coupling constants (*J*) are reported in units of Hertz (Hz). An IRAffinity-1S Fourier transform IR (FTIR) spectrometer (Shimadzu, Tokyo, Japan) was used to record the IR spectra of the compounds. Mass spectra were recorded on an LCMS-8040 tandem mass spectrometer (Shimadzu, Kyoto, Japan) by means of electrospray ionization (ESI). Silica gel 60 F254 thin-layer chromatography (TLC; Merck KGaA, Darmstadt, Germany) was used to control the purity of the obtained compounds.

#### 3.1.1. Synthesis of *N*-(4-Chlorophenyl)hydrazinecarbothioamide (**1**)

An excess of hydrazine hydrate was added to the mixture of 4-chlorophenyl isothiocyanate (0.22 mol, 3.73 g) in ethanol (EtOH; 40 mL) and stirred in an ice bath for 2h. After the reaction was complete, the precipitated product was filtered and washed with cold EtOH. Then, the residue was dried and recrystallized from EtOH [39]. 

#### 3.1.2. Synthesis of 5-((4-Chlorophenyl)amino)-1,3,4-thiadiazole-2-thiol (**2**)

A mixture of *N*-(4-chlorophenyl)hydrazinecarbothioamide (0.02 mol, 4.03 g) and carbon disulfide (0.024 mol, 1.44 mL) was added to EtOH (40 mL) containing NaOH (0.024 mol, 0.96 g) and refluxed for 4 h. The reaction mixture was poured into iced water and acidified by 20% HCl down to pH 4–5, after completion of the reaction. The precipitated product was filtered and washed with water. Then, the residue was dried and recrystallized from EtOH [39].

#### 3.1.3. General Procedure for the Synthesis of Target Compounds (**3a**–**3l**)

Firstly, 5-((4-chlorophenyl)amino)-1,3,4-thiadiazole-2-thiol (**2**) (0.83 mmol), appropriate 2-bromoacetophenone (0.83 mmol), and potassium carbonate (0.83 mmol, 0.11 g) as a catalyst were stirred for 4 h in acetone (20 mL). The acetone was evaporated under reduced pressure after completion of the reaction. The precipitated product was washed with water in order to remove potassium carbonate, before being dried and recrystallized from EtOH [39].

*2-((5-((4-Chlorophenyl)amino)-1,3,4-thiadiazol-2-yl)thio)-1-phenylethan-1-one* (**3a**). Yield: 85%, MP = 208–210 °C, FTIR (ATR, cm^−1^): 3265 (N–H), 2953 (C–H), 1689 (C=O), 684, 748. ^1^H-NMR (300 MHz, DMSO-*d_6_*): δ = 4.97 (2H, s, –CH_2_–), 7.37 (2H, d, *J* = 8.9 Hz, C–H aromatic), 7.58–7.73 (5H, m, C–H aromatic), 8.05 (2H, d, *J* = 8.5 Hz, C–H aromatic), 10.52 (1H, s, –NH). ^13^C-NMR (75 MHz, DMSO-*d_6_*): δ = 41.81, 119.33, 125.80, 128.96, 129.33, 129.39, 134.29, 135.67, 139.68, 153.14, 164.91, 193.62. ESI-MS (*m*/*z*): [M + H]^+^: 362.1 (100%).

*2-((5-((4-Chlorophenyl)amino)-1,3,4-thiadiazol-2-yl)thio)-1-(p-tolyl)ethan-1-one* (**3b**). Yield: 88%, MP = 200–201 °C, FTIR (ATR, cm^−1^): 3248 (N–H), 2891 (C–H), 1687 (C=O), 835. ^1^H-NMR (300 MHz, DMSO-*d_6_*): δ = 2.40 (3H, s, –CH_3_), 4.93 (2H, s, –CH_2_–), 7.37 (4H, d, *J* = 8.9 Hz, C–H aromatic), 7.59 (2H, d, *J* = 8.9 Hz, C–H aromatic), 7.94 (2H, d, *J* = 8.2 Hz, C–H aromatic), 10.51 (1H, s, –NH). ^13^C-NMR (75 MHz, DMSO-*d_6_*): δ = 21.69, 41.77, 119.32, 125.79, 129.09, 129.86, 130.53, 133.17, 139.68, 144.83, 164.90, 193.14. ESI-MS (*m*/*z*): [M + H]^+^: 376.1 (100%).

*2-((5-((4-Chlorophenyl)amino)-1,3,4-thiadiazol-2-yl)thio)-1-(4-methoxyphenyl)ethan-1-one* (**3c**). Yield: 82%, MP = 209–211 °C, FTIR (ATR, cm^−1^): 3263 (N–H), 2966 (C–H), 1681 (C=O), 833. ^1^H-NMR (300 MHz, DMSO-*d_6_*): δ = 3.86 (3H, s, –OCH_3_), 4.90 (2H, s, –CH_2_–), 7.09 (2H, d, *J* = 8.9 Hz, C–H aromatic), 7.38 (2H, d, *J* = 8.9 Hz, C–H aromatic), 7.59 (2H, d, *J* = 8.9 Hz, C–H aromatic), 8.02 (2H, d, *J* = 8.9 Hz C–H aromatic,), 10.51 (1H, s, –NH). ^13^C-NMR (75 MHz, DMSO-*d_6_*): δ = 41.64, 56.13, 114.53, 119.32, 125.79, 128.51, 129.40, 131.39, 139.69, 153.31, 164.07, 164.91, 191.97. ESI-MS (*m*/*z*): [M + H]^+^: 392.1 (100%).

*4-(2-((5-((4-Chlorophenyl)amino)-1,3,4-thiadiazol-2-yl)thio)acetyl)benzonitrile* (**3d**). Yield: 86%, MP = 182–184 °C, FTIR (ATR, cm^−1^): 3244 (N–H), 2906 (C–H), 2229 (C≡N), 1681 (C=O), 833. ^1^H-NMR (300 MHz, DMSO-*d_6_*): δ = 4.90 (2H, s, –CH_2_–), 7.36 (2H, d, *J* = 8.9 Hz, C–H aromatic), 7.58 (2H, d, *J* = 8.9 Hz, C–H aromatic), 8.06 (2H, d, *J* = 8.6 Hz, C–H aromatic), 8.18 (2H, d, *J* = 8.6 Hz, C–H aromatic), 10.52 (1H, s, –NH). ^13^C-NMR (75 MHz, DMSO-*d_6_*): δ = 41.77, 116.06, 118.57, 119.34, 125.84, 129.39, 129.55, 133.34, 138.93, 139.64, 152.69, 165.03, 193.23. ESI-MS (*m*/*z*): [M + H]^+^: 387.1 (100%).

*2-((5-((4-Chlorophenyl)amino)-1,3,4-thiadiazol-2-yl)thio)-1-(4-nitrophenyl)ethan-1-one* (**3e**). Yield: 81%, MP = 197–199 °C, FTIR (ATR, cm^−1^): 3251 (N–H), 2931 (C–H), 1685 (C=O), 1517, 1344 (NO_2_), 852. ^1^H-NMR (300 MHz, DMSO-*d_6_*): δ = 5.01 (2H, s, –CH_2_–), 7.37 (2H, d, *J* = 8.9 Hz, C–H aromatic), 7.58 (2H, d, *J* = 8.9 Hz, C–H aromatic), 8.27 (2H, d, *J* = 8.9 Hz, C–H aromatic), 8.38 (2H, d, *J* = 8.9 Hz, C–H aromatic), 10.52 (1H, s, –NH). ^13^C-NMR (75 MHz, DMSO-*d_6_*): δ = 41.93, 119.34, 124.39, 125.84, 129.39, 130.38, 139.63, 140.40, 150.63, 152.65, 165.04, 193.08. ESI-MS (*m*/*z*): [M + H]^+^: 407.1 (100%).

*2-((5-((4-Chlorophenyl)amino)-1,3,4-thiadiazol-2-yl)thio)-1-(4-fluorophenyl)ethan-1-one* (**3f**). Yield: 79%, MP = 205–207 °C, FTIR (ATR, cm^−1^): 3251 (N–H), 2899 (C–H), 1676 (C=O), 821. ^1^H-NMR (300 MHz, DMSO-*d_6_*): δ = 4.95 (2H, s, –CH_2_–), 7.35–7.44 (4H, m, C–H aromatic), 7.59 (2H, d, *J* = 8.9 Hz, C–H aromatic), 8.14 (2H, dd, *J1* = 5.5 Hz, *J2* = 8.9 Hz, C–H aromatic), 10.51 (1H, s, –NH). ^13^C-NMR (75 MHz, DMSO-*d_6_*): δ = 41.71, 116.39 (*J2* = 21.9 Hz), 119.33, 125.81, 129.39, 132.06 (*J3* = 9.6 Hz), 132.44 (*J4* = 2.6 Hz), 139.67, 153.03, 164.95, 165.80 (*J1* = 251.1 Hz), 192.30. ESI-MS (*m*/*z*): [M + H]^+^: 380.1 (100%).

*1-(4-Chlorophenyl)-2-((5-((4-chlorophenyl)amino)-1,3,4-thiadiazol-2-yl)thio)ethan-1-one* (**3g**). Yield: 75%, MP = 198–200 °C, FTIR (ATR, cm^−1^): 3246 (N–H), 3037 (C–H), 1676 (C=O), 813. ^1^H-NMR (300 MHz, DMSO-*d_6_*): δ = 4.95 (2H, s, –CH_2_–), 7.37 (2H, d, *J* = 8.9 Hz, C–H aromatic), 7.58 (2H, d, *J* = 8.9 Hz, C–H aromatic), 7.65 (2H, d, *J* = 8.6 Hz, C–H aromatic), 8.06 (2H, d, *J* = 8.6 Hz, C–H aromatic), 10.52 (1H, s, –NH). ^13^C-NMR (75 MHz, DMSO-*d_6_*): δ = 41.69, 119.33, 125.81, 129.40, 129.45, 130.89, 134.38, 139.19, 139.66, 153.05, 164.97, 192.78. ESI-MS (*m*/*z*): [M + H]^+^: 396.1 (100%).

*1-(4-Bromophenyl)-2-((5-((4-chlorophenyl)amino)-1,3,4-thiadiazol-2-yl)thio)ethan-1-one* (**3h**). Yield: 84%, MP = 185–187 °C, FTIR (ATR, cm^−1^): 3250 (N–H), 2927 (C–H), 1676 (C=O), 810. ^1^H-NMR (300 MHz, DMSO-*d_6_*): δ = 4.94 (2H, s, –CH_2_–), 7.37 (2H, d, *J* = 8.9 Hz, C–H aromatic ), 7.58 (2H, d, *J* = 8.9 Hz, C–H aromatic), 7.79 (2H, d, *J* = 8.6 Hz, C–H aromatic), 7.97 (2H, d, *J* = 8.6 Hz, C–H aromatic), 10.52 (1H, s, –NH). ^13^C-NMR (75 MHz, DMSO-*d_6_*): δ = 41.66, 119.33, 125.81, 128.44, 129.40, 130.96, 132.40, 134.70, 139.66, 152.93, 164.96, 193.00. ESI-MS (*m*/*z*): [M + H]^+^: 440.0 (100%).

*1-([1,1′-Biphenyl]-4-yl)-2-((5-((4-chlorophenyl)amino)-1,3,4-thiadiazol-2-yl)thio)ethan-1-one* (**3i**). Yield: 89%, MP = 221–223 °C, FTIR (ATR, cm^−1^): 3263 (N–H), 2968 (C–H), 1687 (C=O), 833. ^1^H-NMR (300 MHz, DMSO-*d_6_*): δ = 4.99 (2H, s, –CH_2_–), 7.37 (2H, d, *J* = 8.9 Hz, C–H aromatic), 7.44–7.47 (1H, m, C–H aromatic), 7.52 (2H, t, *J* = 7.5 Hz, C–H aromatic), 7.59 (2H, d, *J* = 8.9 Hz, C–H aromatic), 7.77 (2H, d, *J* = 7.0 Hz, C–H aromatic), 7.87 (2H, d, *J* = 8.5 Hz, C–H aromatic), 8.13 (2H, d, *J* = 8.5 Hz, C–H aromatic), 10.53 (1H, s, –NH). ^13^C-NMR (75 MHz, DMSO-*d_6_*): δ = 41.82, 119.33, 125.81, 127.47, 127.53, 129.02, 129.40, 129.60, 129.73, 134.47, 139.21, 139.68, 145.56, 153.14, 164.94, 193.21. ESI-MS (*m*/*z*): [M + H]^+^: 438.1 (100%).

*2-((5-((4-Chlorophenyl)amino)-1,3,4-thiadiazol-2-yl)thio)-1-(2,4-dimethylphenyl)ethan-1-one* (**3j**). Yield: 77%, MP = 172–174 °C, FTIR (ATR, cm^−1^): 3259 (N–H), 2914 (C–H), 1683 (C=O), 835. ^1^H-NMR (300 MHz, DMSO-*d_6_*): δ = 2.33 (3H, s, –CH_3_), 2.38 (3H, s, –CH_3_), 4.82 (2H, s, –CH_2_–), 7.13–7.18 (2H, m, C–H aromatic), 7.38 (2H, d, *J* = 8.9 Hz, C–H aromatic), 7.59 (2H, d, *J* = 8.9 Hz, C–H aromatic), 7.84 (1H, d, *J* = 7.7 Hz, C–H aromatic), 10.55 (1H, s, –NH). ^13^C-NMR (75 MHz, DMSO-*d_6_*): δ = 21.40, 21.41, 43.78, 119.32, 125.76, 126.90, 129.39, 130.25, 132.97, 133.41, 138.73, 139.74, 142.75, 153.21, 164.87, 196.28. ESI-MS (*m*/*z*): [M + H]^+^: 390.1 (100%).

*2-((5-((4-Chlorophenyl)amino)-1,3,4-thiadiazol-2-yl)thio)-1-(2,4-difluorophenyl)ethan-1-one* (**3k**). Yield: 80%, MP = 203–204 °C, FTIR (ATR, cm^−1^): 3248 (N–H), 2914 (C–H), 1680 (C=O), 829. ^1^H-NMR (300 MHz, DMSO-*d_6_*): δ = 4.82 (2H, s, –CH_2_–), 7.25–7.32 (1H, m, C–H aromatic), 7.37 (2H, d, *J* = 8.9 Hz, C–H aromatic), 7.46–7.54 (1H, m, C–H aromatic), 7.59 (2H, d, *J* = 8.9 Hz, C–H aromatic), 7.97–8.05 (1H, m, C–H aromatic), 10.52 (1H, s, –NH). ^13^C-NMR (75 MHz, DMSO-*d_6_*): δ = 44.92 (d, *J* = 7.3 Hz), 105.83 (t, *J* = 26.6 Hz), 113.07 (dd, *J1* = 3.0 Hz, *J2* = 21.6 Hz), 119.33, 121.22 (dd, *J1* = 3.3 Hz, *J2* = 12.1 Hz), 125.82, 129.39, 133.38 (dd, *J1* = 3.5 Hz, *J2* = 10.9 Hz), 139.65, 152.83, 162.54 (d, *J1* = 13.2 Hz, *J2* = 256.4 Hz), 164.91, 165.94 (dd, *J1* = 12.9 Hz, *J2* = 253.5 Hz), 190.18 (d, *J* = 4.25 Hz). ESI-MS (*m*/*z*): [M + H]^+:^ 398.1 (100%).

*2-((5-((4-Chlorophenyl)amino)-1,3,4-thiadiazol-2-yl)thio)-1-(2,4-dichlorophenyl)ethan-1-one* (**3l**). Yield: 78%, MP = 176–178 °C, FTIR (ATR, cm^−1^): 3248 (N–H), 2914 (C–H), 1660 (C=O), 831. ^1^H-NMR (300 MHz, DMSO-*d_6_*): δ = 4.80 (2H, s, –CH_2_–), 7.38 (2H, d, *J* = 8.9 Hz, C–H aromatic), 7.59 (2H, d, *J* = 8.9 Hz, C–H aromatic), 7.62 (1H, dd, *J1* = 2.0 Hz, *J2* = 8.3 Hz, C–H aromatic) 7.78 (1H, d, *J* = 2.0 Hz, C–H aromatic), 7.87 (1H, d, *J* = 8.3 Hz, C–H aromatic), 10.53 (1H, s, –NH). ^13^C-NMR (75 MHz, DMSO-*d_6_*): δ = 43.85, 119.35, 125.85, 128.07, 129.42, 130.67, 131.91, 132.19, 135.62, 137.32, 139.64, 152.58, 165.02, 194.77. ESI-MS (*m*/*z*): [M + H]^+^: 430.0 (100%). 

### 3.2. Antifungal Assay

The anticandidal effects of compounds **3a**–**3l** were screened according to the method of EUCAST determinant (EDef 7.1) [40]. The antifungal assays were performed as previously described [28]. Fluconazole was used as a reference drug. The in vitro growth inhibitory activity of the compounds was tested against *Candida albicans* (ATCC 90028), *Candida crusei* (ATCC 6258), *Candida parapsilosis* (ATCC 22019), *Candida tropicalis* (ATCC 13803), *Candida albicans* (ATCC 10231), *Candida glabrata* (ATCC 2001), *Candida famata* (Abant İzzet Baysal University Medical Faculty Hospital, clinical isolate), and *Candida lusitaniae* (Abant İzzet Baysal University Medical Faculty Hospital, clinical isolate). Results for MIC values were determined in duplicate for each compound. Fungal strains incubated overnight at 37 °C were maintained in Roswell Park Memorial Institute (RPMI) medium. Adjustment of the inoculum density to a 0.5 McFarland turbidity standard was performed by means of a spectrophotometer. The obtained inoculum suspension included 0.5 × 10^5^ to 2.5 × 10^5^ colony-forming units (cfu)/mL for fungi. The test, in which the two-fold serial dilution technique was applied, was performed in RPMI at pH 7. The last well on the microplates, which contained only the inoculated broth, was kept as control, and the last well with no growth of microorganism was recorded to represent the minimum inhibitory concentration (MIC_50_) in μg/mL. DMSO was used as a solvent. Further dilutions of the standard drug and obtained compounds in the RPMI medium were carried out in the required doses at concentrations of 160–0.312 μg/mL. After a 24-h incubation of the final plates containing microorganism strains, MIC_50_ values were determined using a microplate reader at 490 nm. Each experiment was performed twice in the antifungal assay.

### 3.3. Quantification of Ergosterol Level

Total sterols were extracted from *C. albicans* ATCC 10231, which was licensed by Breivik and Owades to the literature [33]. Quantification of ergosterol levels in this extract was performed as previously described [41]. In order to inoculate, 50 mL of Sabouraud dextrose broth (Difco) including 2.5, 5, 10, 20, and 40 µg/mL of compounds **3k**, **3l** and fluconazole utilized a single *C. albicans* colony from an overnight Sabouraud dextrose agar plate culture. The cultures were incubated for 16 h with shaking at 35 °C. The stationary-phase cells were taken by centrifugation at 2700 rpm (Hettich, Rotina 380 R, Germany) for 5 min and washed once with sterile distilled water. Three milliliters of 25% alcoholic potassium hydroxide solution was added to each pellet and vortex mixed for 1 min. Cell suspensions were carried to sterile borosilicate glass screw-cap tubes and were incubated in an 85 °C water bath for 1 h. After this stage, tubes were allowed to cool to room temperature. Sterols were then extracted by addition of a mixture of 1 mL of sterile distilled water and 3 mL of chloroform followed by vigorous vortex mixing for 3 min. The chloroform layer was transferred to a clean borosilicate glass screw-cap tube and 1 µL of sterol extract was injected into the LC–MS/MS system (Shimadzu LCMS 8040, Kyoto, Japan). The ergosterol quantity in the negative control samples was regarded as 100%. All concentrations were analyzed in quadruplicate, and the results were expressed as means ± standard deviation (SD).

### 3.4. Molecular Docking Studies

Molecular docking studies were performed using an in silico procedure to define the binding modes of compounds **3k** and **3l** in the 14-α-sterol demethylase enzyme active region. X-ray crystal structures of 14-α-sterol demethylase (PDB code: 5FSA) [34] were retrieved from the Protein Data Bank server (www.pdb.org). The docking procedure was applied using the Schrödinger Maestro [42] interface as in previously described by our research group [43].

The structure of the ligand was built using the *Schrödinger Maestro* [42] interface and was then submitted to the *Protein Preparation Wizard* protocol of the *Schrödinger Suite 2016 Update 2* [44]. The ligands were prepared using *LigPrep 3.8* [45] to correctly assign the protonation states at pH 7.4 ± 1.0, as well as the atom types. Bond orders were assigned and hydrogen atoms were added to the structures. The grid generation was formed using the *Glide 7.1* [46] program and docking runs were performed with standard precision docking mode (SP).

### 3.5. Theoretical Determination of ADME Properties

Physicochemical parameters of obtained compounds (**3a**–**3l**) were evaluated using QikProp 4.8 [36]. 

## 4. Conclusions 

In this study, we synthesized a series of new 1,3,4-thiadiazole derivatives to evaluate their possible antifungal properties against different fungal strains. The preliminary results indicated that final compounds **3a**–**3l** showed antifungal activities to different extents. In the series, compounds **3k** and **3l** displayed excellent antifungal activities against all of the tested fungi. In particular, compound **3l** was the most effective derivative against *C. albicans* ATCC 10231. Compounds **3k** and **3l** possess significant activities due to the fluoro and chloro groups at the second position of the phenyl moiety. In addition to their good antifungal activity, all compounds in the series exhibited a good predicted pharmacokinetics profile. Furthermore, a primary mechanism-of-action study showed that the antifungal activity of compounds **3k** and **3l** was related to the inhibition of ergosterol biosynthesis in *C. albicans.* Moreover, in the docking study, significant interactions were observed between compounds **3k** and **3l** and 14-α-sterol demethylase, which is a key enzyme in ergosterol biosynthesis. Consequently, the present study introduced new 1,3,4-thiadiazole derivatives as potent antifungal candidates, which may assist scientists in the synthesis of more active compounds with a similar structure.

## Figures and Tables

**Table:** 

Compound	R_1_	R_2_
**3a**	–H	–H
**3b**	–H	–CH_3_
**3c**	–H	–OCH_3_
**3d**	–H	–CN
**3e**	–H	–NO_2_
**3f**	–H	–F
**3g**	–H	–Cl
**3h**	–H	–Br
**3i**	–H	–Phenyl
**3j**	–CH_3_	–CH_3_
**3k**	–F	–F
**3l**	–Cl	–Cl

**Table 1:** Minimum inhibitory concentration (MIC_50_; µg/mL) values of compounds on eight strains of *Candida* (**3a**–**3l**).

Comp.	1	2	3	4	5	6	7	8
**3a**	40	40	20	20	20	20	20	80
**3b**	40	20	20	20	20	20	20	10
**3c**	40	20	40	40	20	20	40	20
**3d**	80	20	20	20	40	20	80	40
**3e**	80	80	80	80	80	80	80	80
**3f**	40	20	20	20	20	20	20	20
**3g**	20	20	20	20	20	20	20	20
**3h**	20	20	10	20	20	20	40	20
**3i**	40	40	40	40	40	40	40	40
**3j**	40	80	20	>160	40	40	40	40
**3k**	10	10	10	20	10	20	10	10
**3l**	10	10	10	20	5	10	10	10
**Fluconazole**	10	20	10	20	20	20	20	10

**1.***Candida albicans* ATCC 90028, **2.** *Candida crusei* ATCC 6258, **3**. *Candida parapsilosis* ATCC 22019, **4**. *Candida tropicalis* ATCC 13803, **5**. *Candida albicans* ATCC 10231, **6**. *Candida glabrata* ATCC 2001, **7**. *Candida famata* (Abant İzzet Baysal University Medical Faculty Hospital, clinic isolate), **8**. *Candida lusitaniae* (Abant İzzet Baysal University Medical Faculty Hospital, clinic isolate).

**Table 2:** Percentage ergosterol level of *C. albicans* (ATCC 10231) after treatment with compounds **3k** and **3l** and fluconazole.

Compound	2.5 µg/mL	5 µg/mL	10 µg/mL	20 µg/mL	40 µg/mL
**3k**	45.38 ± 1.75	38.17 ± 1.21	36.24 ± 1.17	28.33 ± 1.12	20.97 ± 0.88
**3l**	39.21 ± 1.24	33.24 ± 1.18	26.55 ± 0.97	21.74 ± 0.82	17.29 ± 0.64
**Fluconazole**	77.82 ± 1.43	58.02 ± 1.12	44.82 ± 1.24	30.65 ± 1.11	18.27 ± 0.72

**Table 3:** Calculated absorption, distribution, metabolism, and excretion (ADME) parameters of compounds **3a**–**3l**.

Compound	MW	RB	DM	MV	DHB	AHB	PSA	LogP	LogS	PCaco	PM	%HOA	VRF	VRT
**3a**	361.86	6	5.741	1067.2	1	4.5	68.51	4.233	−5.731	797.651	2	100	0	1
**3b**	375.89	6	6.021	1126.1	1	4.5	68.51	4.537	−6.293	797.651	3	100	0	1
**3c**	391.89	7	6.627	1133.6	1	5.25	78.875	4.247	−5.796	794.837	3	100	0	1
**3d**	386.87	7	6.187	1133.9	1	6	94.305	3.48	−6.702	164.955	2	87.011	0	1
**3e**	406.86	7	8.43	1149.6	1	5.5	117.18	3.538	−5.987	81.982	3	81.911	0	1
**3f**	379.85	6	5.193	1083.3	1	4.5	68.514	4.469	−6.096	797.555	2	100	0	1
**3g**	396.31	6	5.161	1111.3	1	4.5	68.513	4.727	−6.472	797.588	2	100	0	1
**3h**	440.76	6	5.241	1120.2	1	4.5	68.513	4.804	−6.587	797.605	2	100	0	1
**3i**	437.96	7	8.053	1305.8	1	4.5	70.177	5.862	−7.79	742.617	2	100	1	1
**3j**	389.92	6	4.88	1175.2	1	4.5	66.349	4.78	−6.611	813.51	4	100	0	1
**3k**	397.84	6	5.396	1072.2	1	4.5	69.187	4.371	−5.216	765.672	2	100	0	0
**3l**	430.75	6	5.548	1158.7	1	4.5	64.786	5.318	−6.907	1112.803	2	100	1	1

**MW:** molecular weight; **RB:** number of rotatable bonds; **DM:** computed dipole moment; **MV:** total solvent-accessible volume; **DHB:** estimated number of hydrogen-bond donors; **AHB:** estimated number of hydrogen-bond acceptors; **PSA:** van der Waals surface area of polar nitrogen and oxygen atoms and carbonyl carbon atoms; **LogP:** predicted octanol/water partition coefficient; **LogS:** predicted aqueous solubility; **PCaco:** predicted apparent Caco-2 cell permeability; **PM:** number of likely metabolic reactions; **%HOA:** predicted human oral absorption percentage; **VRF:** number of violations of Lipinski’s rule of five (the rules are as follows: MW < 500, logP < 5, DHB ≤ 5, AHB ≤ 10, positive PSA value); **VRT:** number of violations of Jorgensen’s rule of three (the rules are as follows: logS > −5.7, PCaco > 22 nm/s, PM < 7).

## Supplementary Materials

The supplementary materials are available online.
