# Supplementary material for: Synthesis and Evaluation of New 1,3,4-Thiadiazole Derivatives as Potent Antifungal Agents
Source: Molecules. 2018 Nov 29;23(12):3129. doi: 10.3390/molecules23123129 (PMC6321371; doi:10.3390/molecules23123129)
Supplement: Supplementary file 1 [file molecules-23-03129-s001.pdf]

SUPPORTING INFORMATION

Synthesis and Molecular Docking Studies of 1,3,4-Thiadiazole Derivatives as Potent Antifungal Agents

Ahmet Çağrı Karaburun[1], Ulviye Acar Çevik[1,2*], Derya Osmaniye[1,2], Begüm Nurpelin Sağlık[1,2], Betül Kaya Çavuşoğlu[1], Serkan Levent[1,2], Yusuf Özkay[1,2], Ali Savaş Koparal[3], Mustafa Behçet[4], Zafer Asım Kaplancıklı[1]

[1]Department of Pharmaceutical Chemistry, Faculty of Pharmacy, Anadolu University, Eskişehir, Turkey
[2]Doping and Narcotic Compounds Analysis Laboratory, Faculty of Pharmacy, Anadolu University, Eskişehir, Turkey
[3]Open Education Faculty, Anadolu University, Eskişehir, Turkey
[4]Department of Medical Microbiology, Faculty of Medicine, Abant İzzet Baysal University, Bolu, Turkey

* Corresponding author.

E-mail address: uacar@anadolu.edu.tr

Tel: +90-222-3350580/ *Fax*: +90-222-3350750.

Address: Anadolu University, Faculty of Pharmacy, Department of Pharmaceutical Chemistry, 26470, Eskişehir, Turkey.

DOPNALAB

Item	Value
Acquired Date&Time	2.07.2018 10:24:51
Acquired by	System Administrator
Filename	C:\Users\dopnalab\Desktop\derya\kt seris\kt-11.ispd
Spectrum name	kt-11
Sample name	KT-1
Sample ID	
Option	
Comment	
No. of Scans	10
Resolution	4 [cm-1]
Apodization	Happ-Genzel

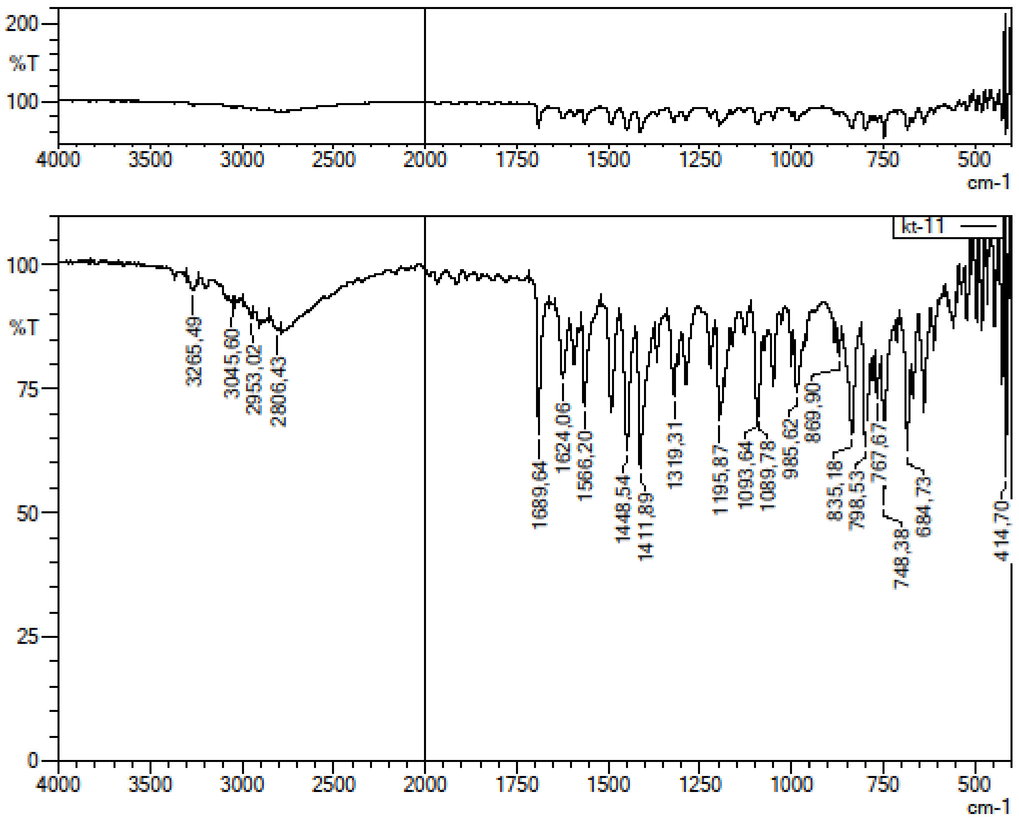

Spectra 1. IR spectra of compound **3a**

LCMSMS ANALYSES REPORT

Sample Name	:KT-1		
Sample ID	:		
Data Filename	: KT-1_derya _024.lcd		
Method Filename	: genel.lcm		
Batch Filename	: batch.lcb		
Vial #	: 1-46		
Injection Volume	: 0,3 uL	Sample Type	: Unknown
Date Acquired	: 22.11.2017 20:25:48	Acquired by	: System Administrator
Date Processed	: 22.11.2017 20:27:49	Processed by	: System Administrator

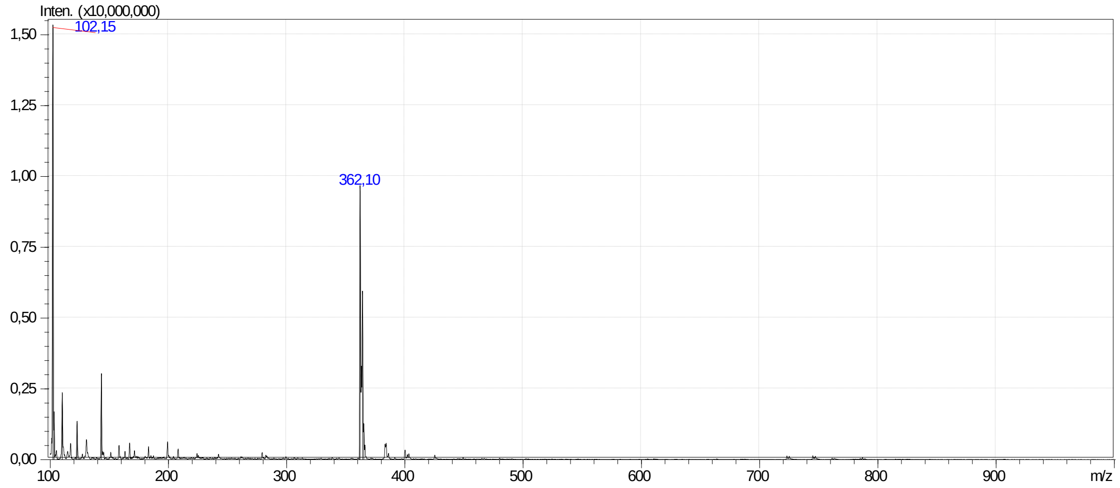

[MS Spectrum]

# of Peaks 8

Raw Spectrum [0,034->0,440],(scan:[3->27])

Background No Background Spectrum

Base Peak m/z 102,15 (Inten : 15.253.587)

m/z	Absolute Intensity	Relative Intensity
102,15	15253587	100,00
103,20	1695722	11,12
110,10	2373594	15,56
122,65	1337398	8,77
143,20	3041542	19,94
362,10	9679961	63,46
364,10	5950120	39,01

Spectra 2. LCMSMS spectra of compound **3a**

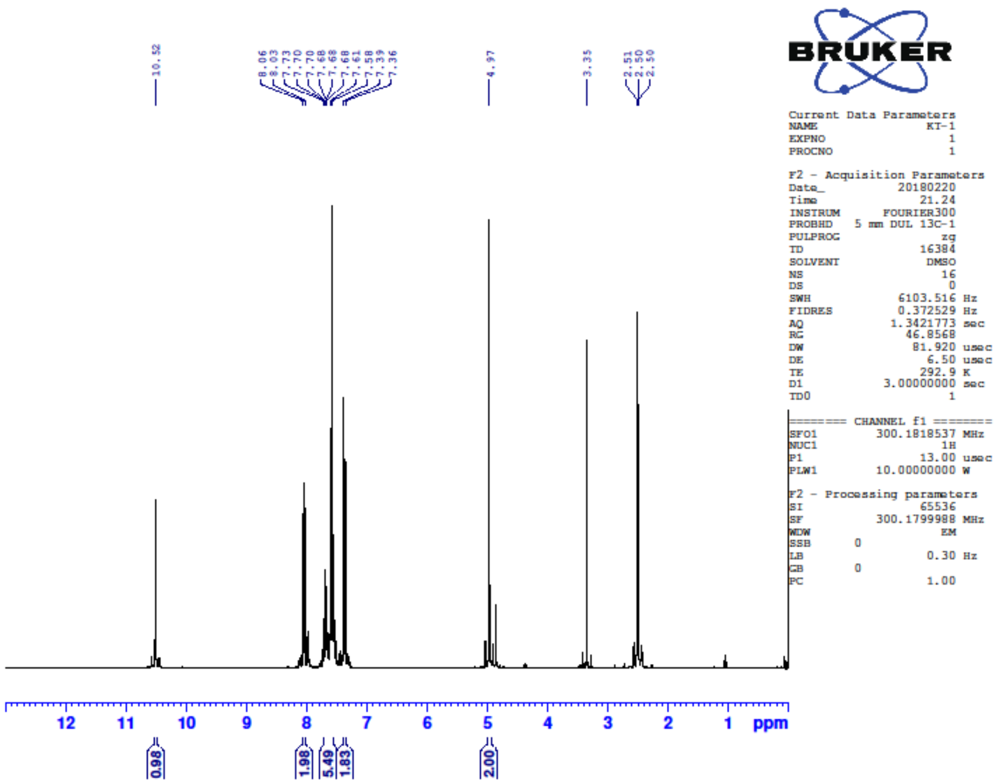

Spectra 3. ^{1}H-NMR spectra of compound **3a**

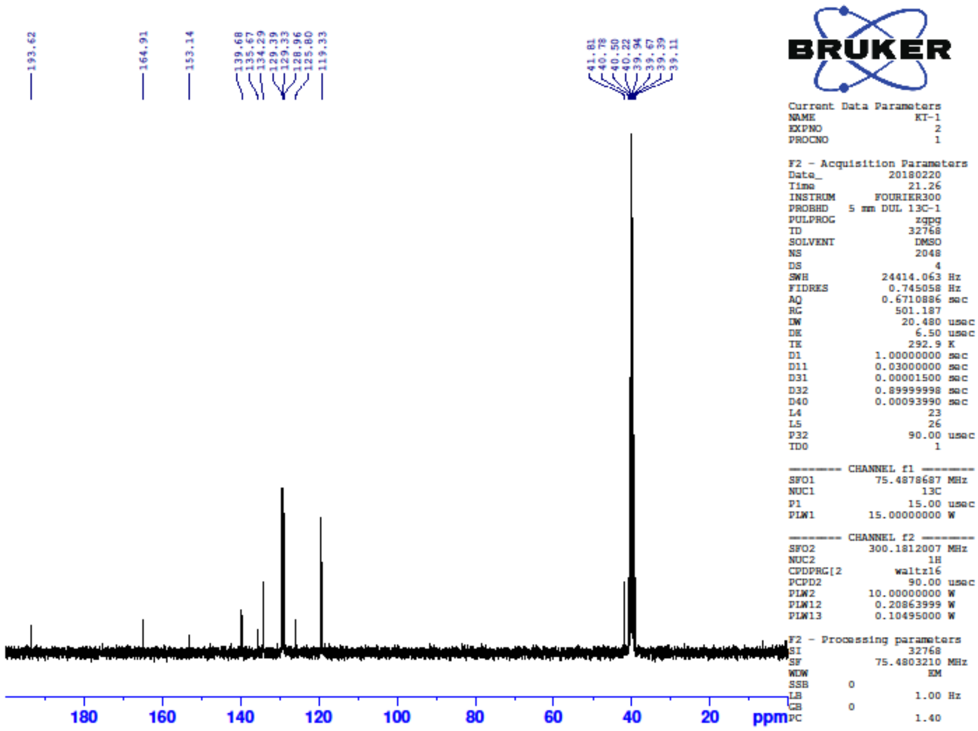

Spectra 4. ^{13}C-NMR spectra of compound **3a**

DOPNALAB

	Item	Value
	Acquired Date&Time	2.07.2018 10:28:12
	Acquired by	System Administrator
	Filename	C:\Users\dopnalab\Desktop\derya\kt seris\kt-21.ispd
	Spectrum name	kt-21
	Sample name	KT-2
	Sample ID	
	Option	
	Comment	
	No. of Scans	10
	Resolution	4 [cm-1]
	Apodization	Happ-Genzel

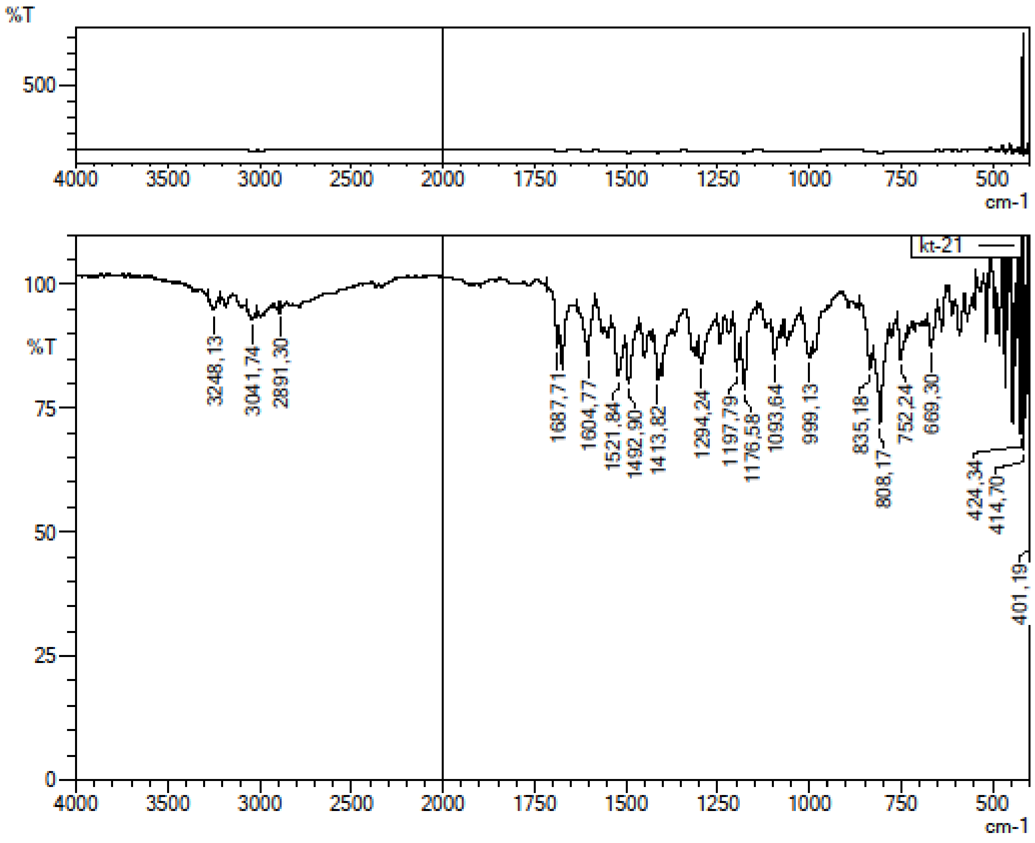

Spectra 5. IR spectra of compound **3b**

LCMSMS ANALYSES REPORT

Sample Name	:KT-2		
Sample ID	:		
Data Filename	: KT-2_derya _025.lcd		
Method Filename	: genel.lcm		
Batch Filename	: batch.lcb		
Vial #	: 1-47		
Injection Volume	: 0,3 uL	Sample Type	: Unknown
Date Acquired	: 22.11.2017 20:28:23	Acquired by	: System Administrator
Date Processed	: 22.11.2017 20:30:24	Processed by	: System Administrator

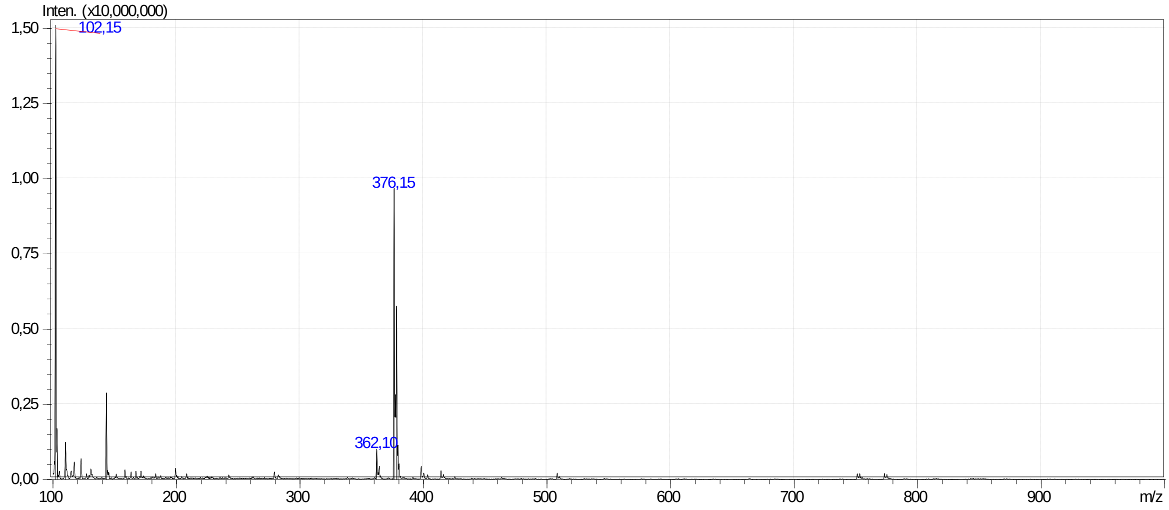

[MS Spectrum]

# of Peaks 8

Raw Spectrum [0,034->0,440],(scan:[3->27])

Background No Background Spectrum

Base Peak m/z 102,15 (Inten : 14.990.989)

m/z	Absolute Intensity	Relative Intensity
102,15	14990989	100,00
103,25	1645699	10,98
110,10	1239613	8,27
143,20	2879617	19,21
362,10	1011352	6,75
376,15	9673877	64,53
378,10	5769623	38,49

Spectra 6. LCMSMS spectra of compound **3b**

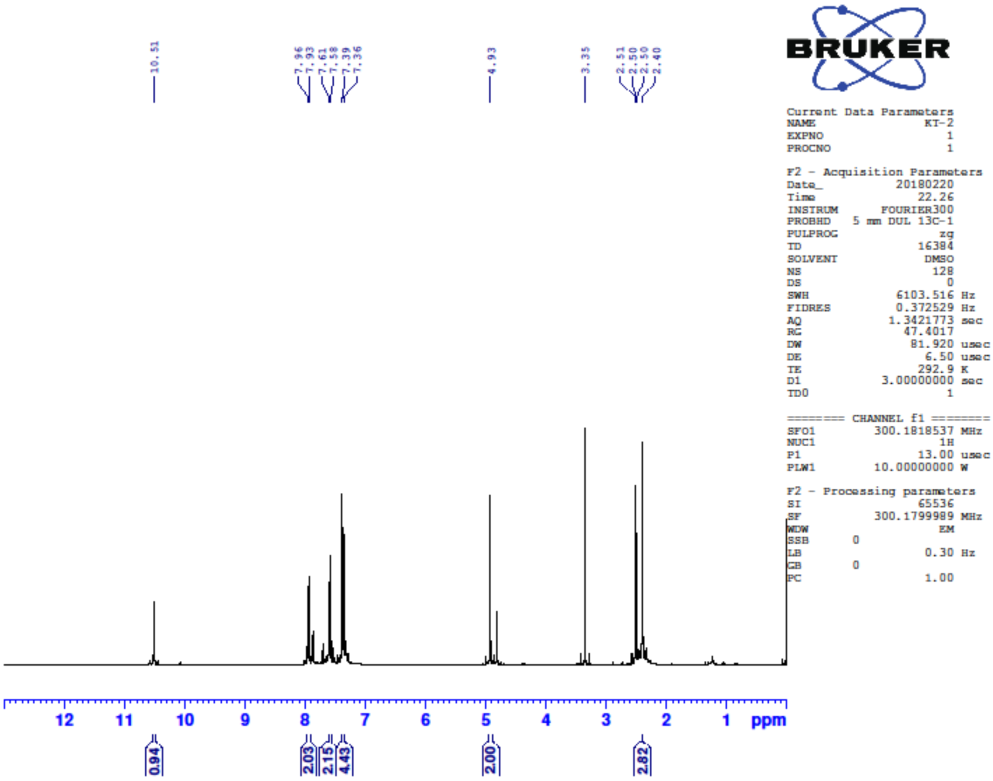

Spectra 7. ^{1}H-NMR spectra of compound **3b**

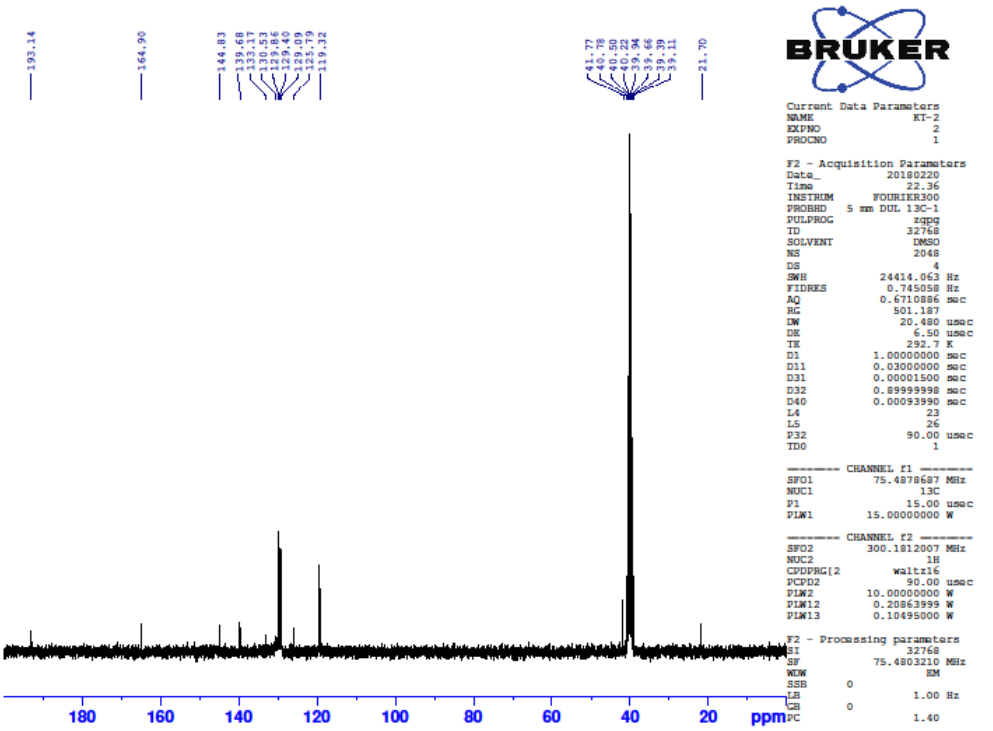

Spectra 8. ^{13}C-NMR spectra of compound **3b**

DOPNALAB

	Item	Value
	Acquired Date&Time	2.07.2018 10:30:41
	Acquired by	System Administrator
	Filename	C:\Users\dopnalab\Desktop\derya\kt sens\kt-31.ispd
	Spectrum name	kt-31
	Sample name	KT-3
	Sample ID	
	Option	
	Comment	
	No. of Scans	10
	Resolution	4 [cm-1]
	Apodization	Happ-Genzel

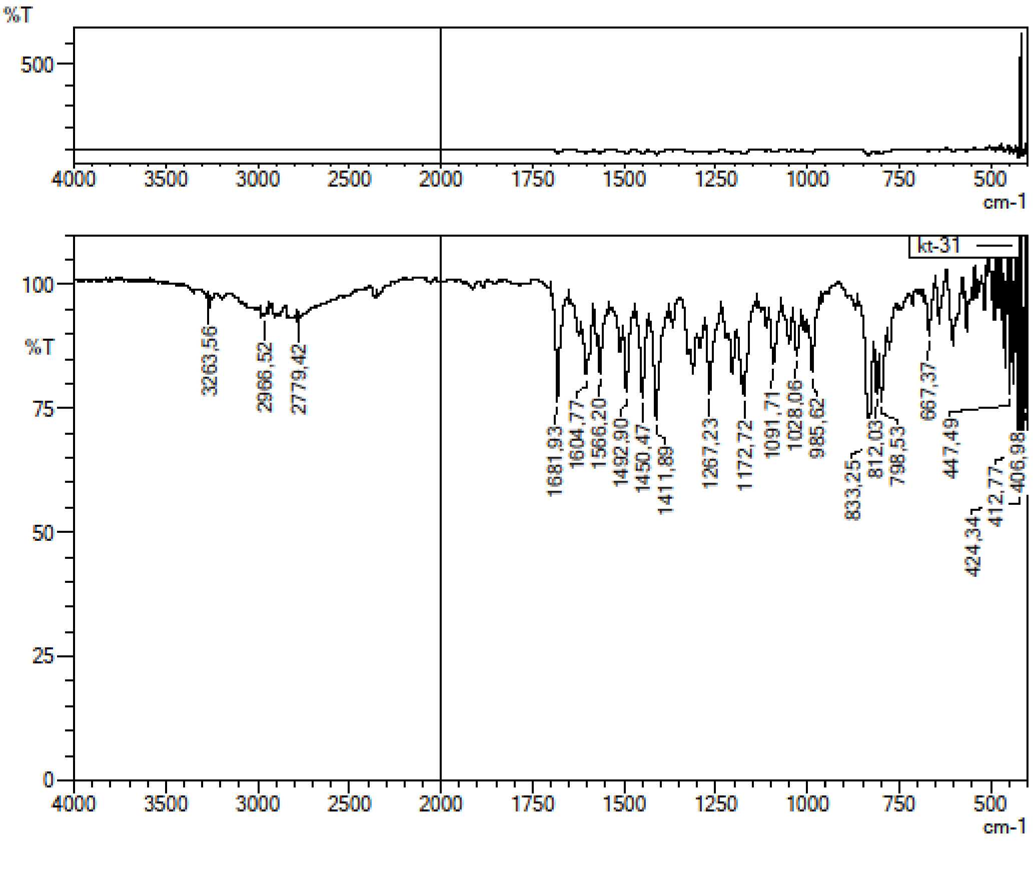

Spectra 9. IR spectra of compound **3c**

LCMSMS ANALYSES REPORT

Sample Name	:KT-3		
Sample ID	:		
Data Filename	: KT-3_derya _026.lcd		
Method Filename	: genel.lcm		
Batch Filename	: batch.lcb		
Vial #	: 1-48		
Injection Volume	: 0,3 uL	Sample Type	: Unknown
Date Acquired	: 22.11.2017 20:31:01	Acquired by	: System Administrator
Date Processed	: 22.11.2017 20:33:03	Processed by	: System Administrator

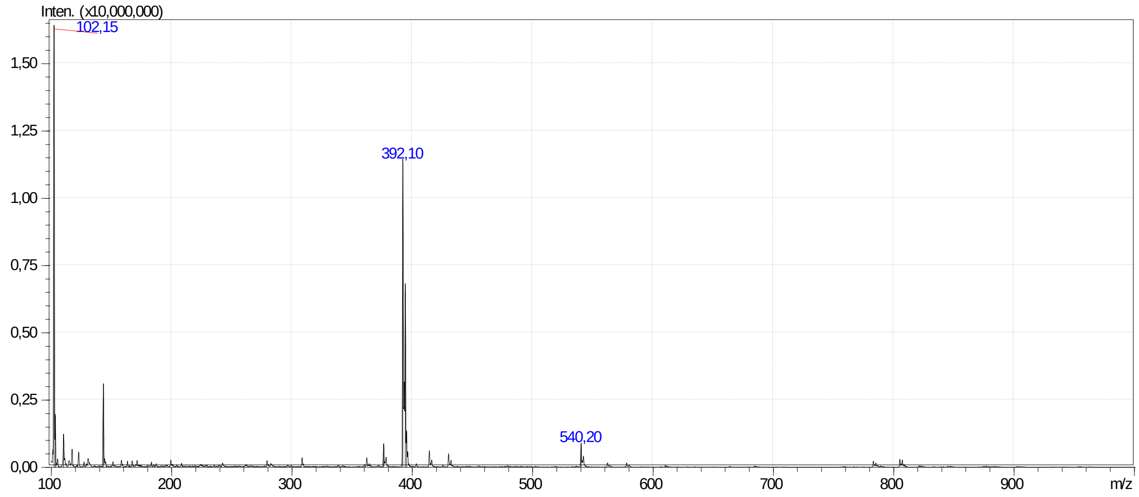

[MS Spectrum]

# of Peaks 9

Raw Spectrum [0,034->0,372],(scan:[3->23])

Background No Background Spectrum

Base Peak m/z 102,15 (Inten : 16.292.742)

m/z	Absolute Intensity	Relative Intensity
102,15	16292742	100,00
103,25	1921242	11,79
110,15	1228544	7,54
143,20	3113847	19,11
376,15	874332	5,37
392,10	11450254	70,28
394,10	6833743	41,94

Spectra 10. LCMSMS spectra of compound **3c**

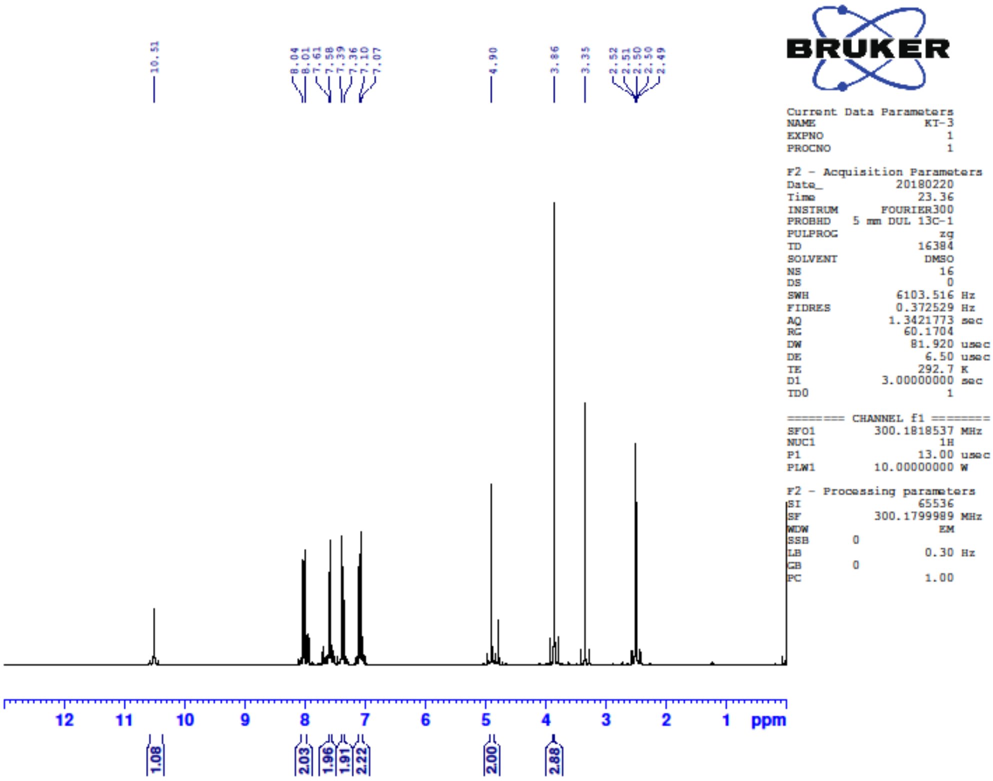

Spectra 11. ^{1}H-NMR spectra of compound **3c**

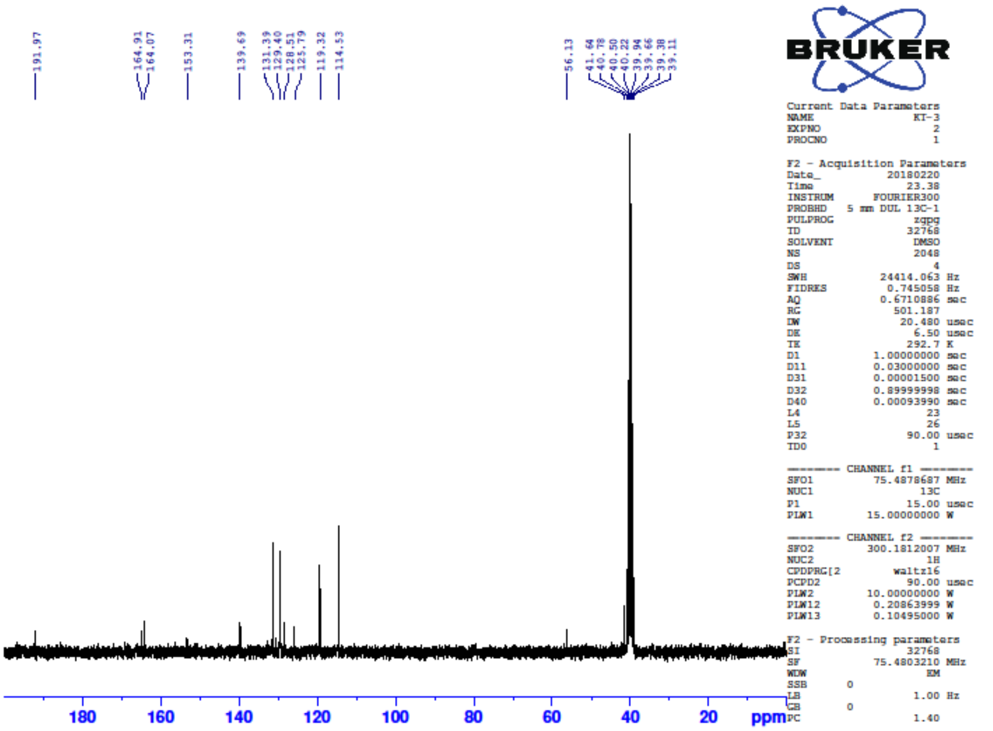

Spectra 12. ^{13}C-NMR spectra of compound **3c**

DOPNALAB

	Item	Value
	Acquired Date&Time	2.07.2018 10:33:53
	Acquired by	System Administrator
	Filename	C:\Users\dopnalab\Desktop\derya\kt seris\kt-41.ispd
	Spectrum name	kt-41
	Sample name	KT-4
	Sample ID	
	Option	
	Comment	
	No. of Scans	10
	Resolution	4 [cm-1]
	Apodization	Happ-Genzel

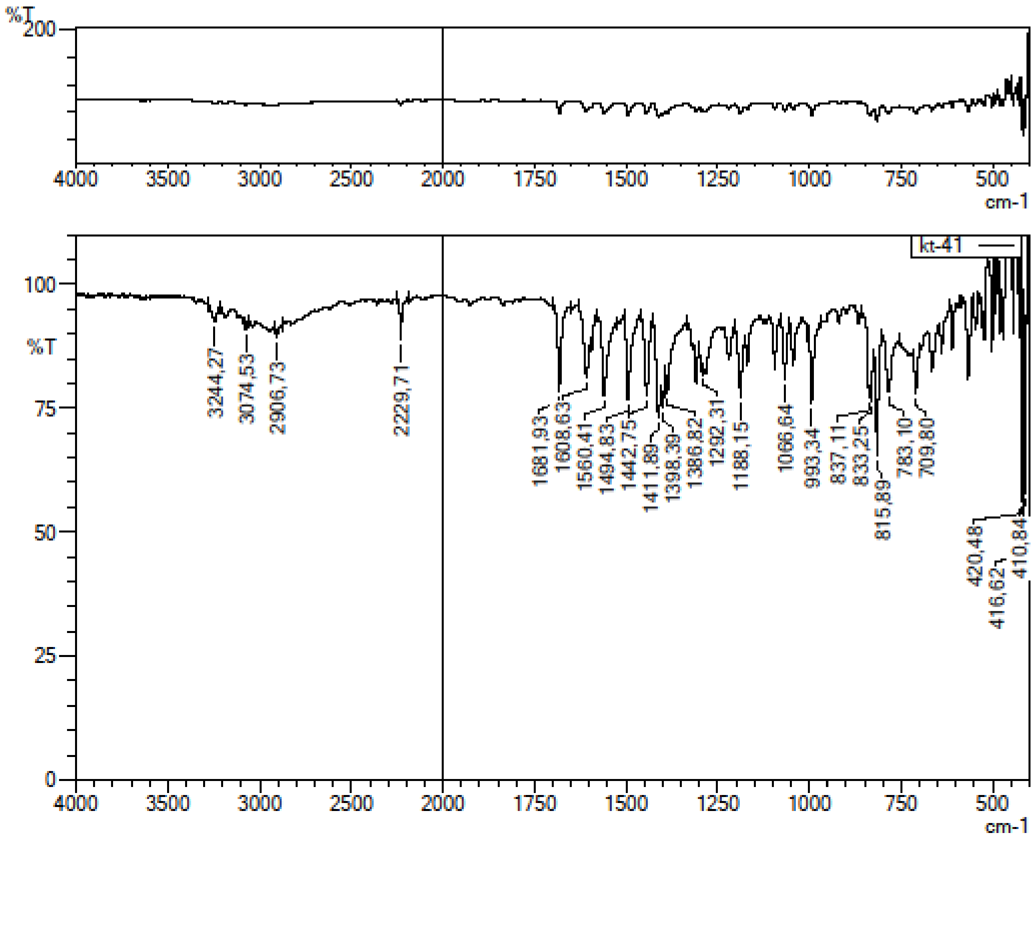

Spectra 13. IR spectra of compound **3d**

LCMSMS ANALYSES REPORT

Sample Name	:KT-4		
Sample ID	:		
Data Filename	: KT-4_derya _027.lcd		
Method Filename	: genel.lcm		
Batch Filename	: batch.lcb		
Vial #	: 1-49		
Injection Volume	: 0,3 uL	Sample Type	: Unknown
Date Acquired	: 22.11.2017 20:33:35	Acquired by	: System Administrator
Date Processed	: 22.11.2017 20:35:37	Processed by	: System Administrator

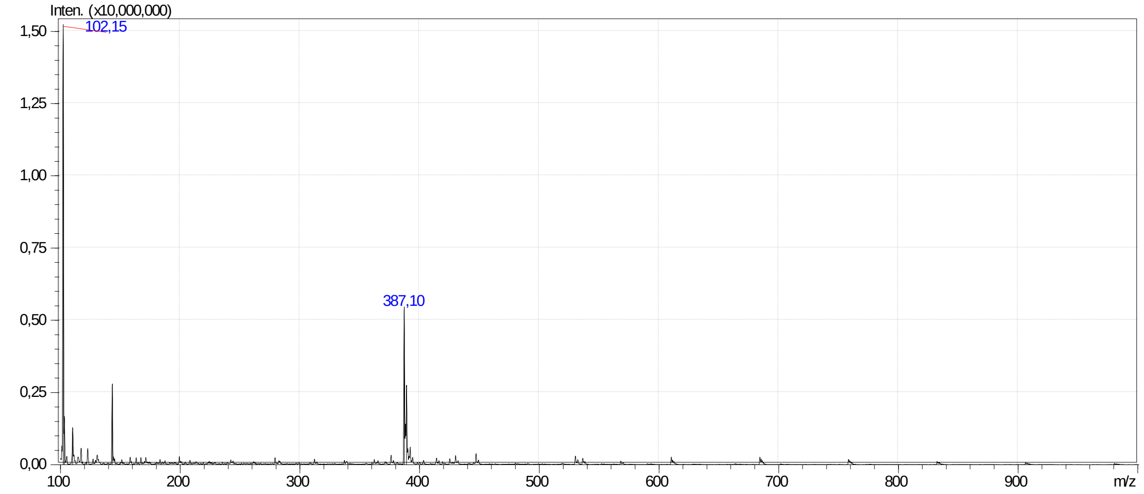

[MS Spectrum]

# of Peaks	6
Raw Spectrum	[0,034->0,440],(scan:[3->27])
Background	No Background Spectrum
Base Peak	m/z 102,15 (Inten : 15.173.742)

m/z	Absolute Intensity	Relative Intensity
102,15	15173742	100,00
103,20	1678239	11,06
110,10	1284762	8,47
143,20	2797350	18,44
387,10	5465156	36,02

Spectra 14. LCMSMS spectra of compound **3d**

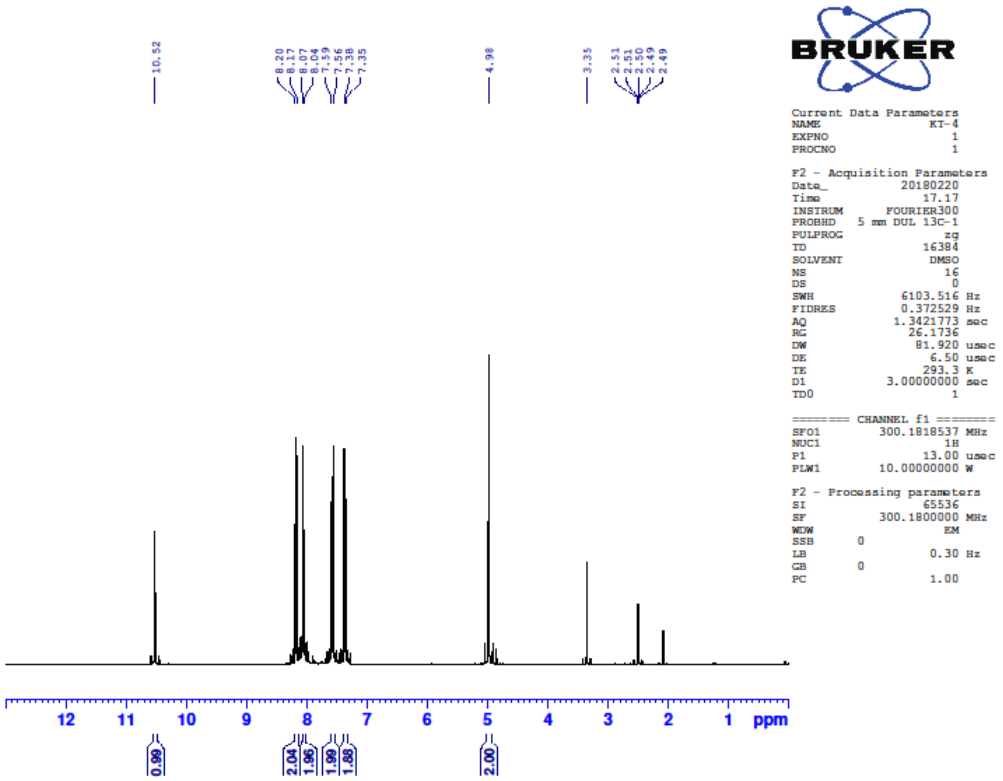

Spectra 15. ^{1}H-NMR spectra of compound **3d**

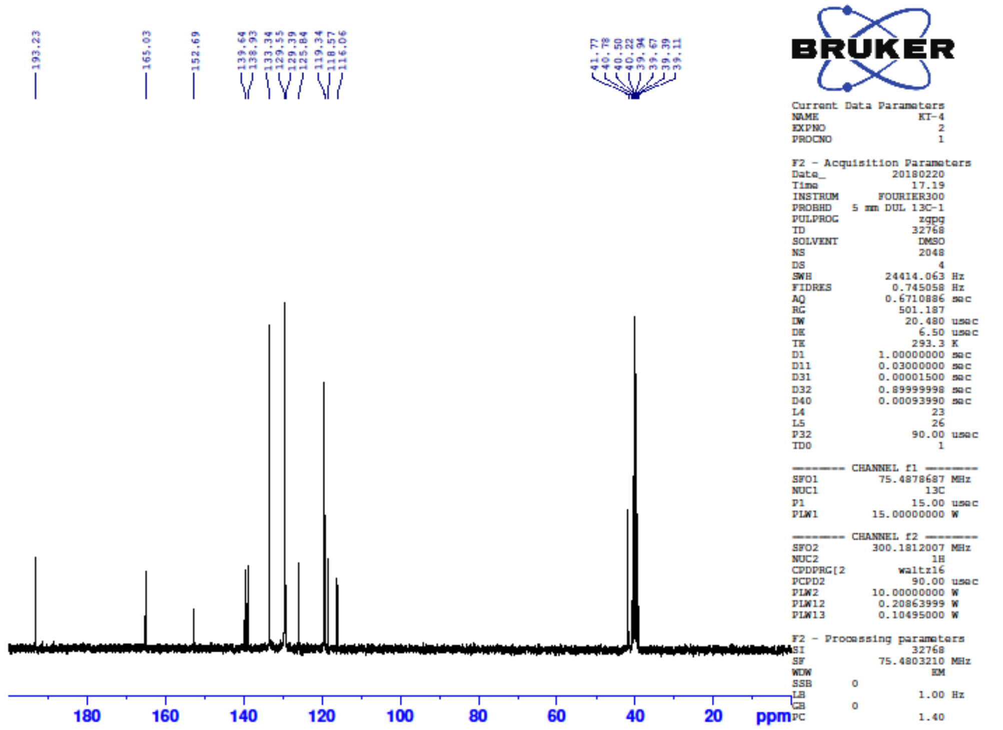

Spectra 16. ^{13}C-NMR spectra of compound **3d**

DOPNALAB

	Item	Value
	Acquired Date&Time	2.07.2018 10:40:43
	Acquired by	System Administrator
	Filename	C:\Users\dopnalab\Desktop\derya\kt seris\kt-51.ispd
	Spectrum name	kt-51
	Sample name	KT-5
	Sample ID	
	Option	
	Comment	
	No. of Scans	10
	Resolution	4 [cm-1]
	Apodization	Happ-Genzel

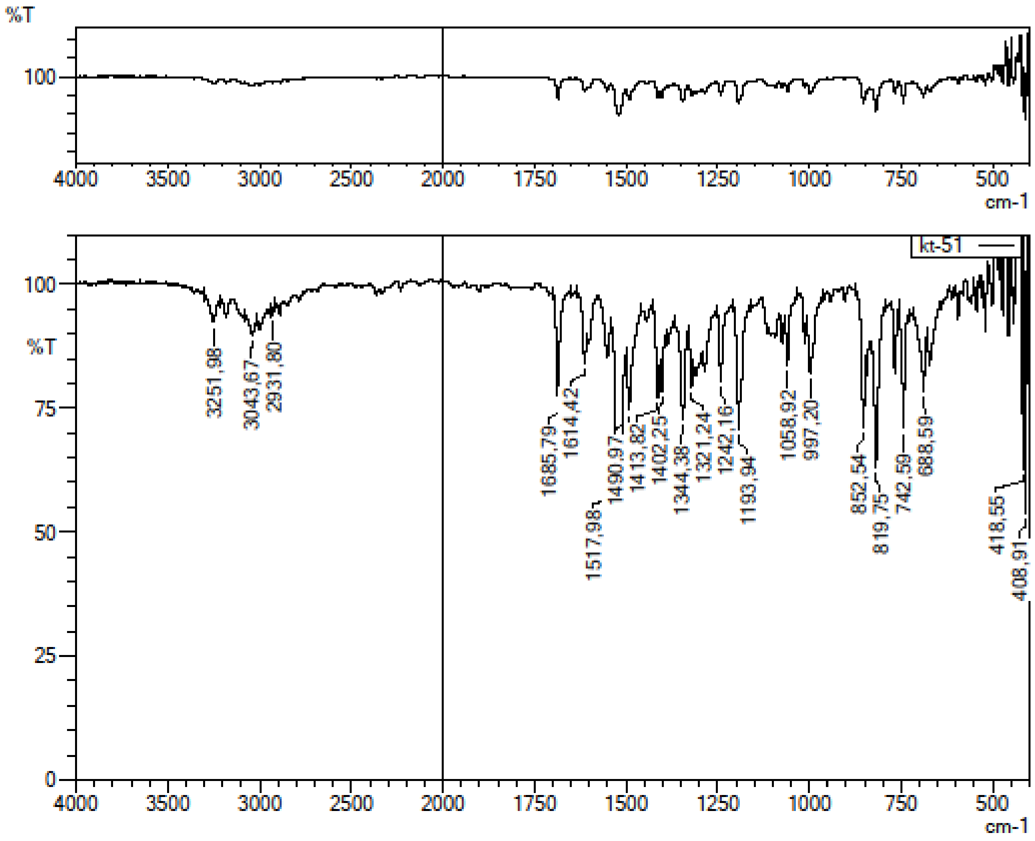

Spectra 17. IR spectra of compound **3e**

LCMSMS ANALYSES REPORT

Sample Name	:KT-5		
Sample ID	:		
Data Filename	: KT-5_derya _028.lcd		
Method Filename	: genel.lcm		
Batch Filename	: batch.lcb		
Vial #	: 1-50		
Injection Volume	: 0,3 uL	Sample Type	: Unknown
Date Acquired	: 22.11.2017 20:36:13	Acquired by	: System Administrator
Date Processed	: 22.11.2017 20:38:15	Processed by	: System Administrator

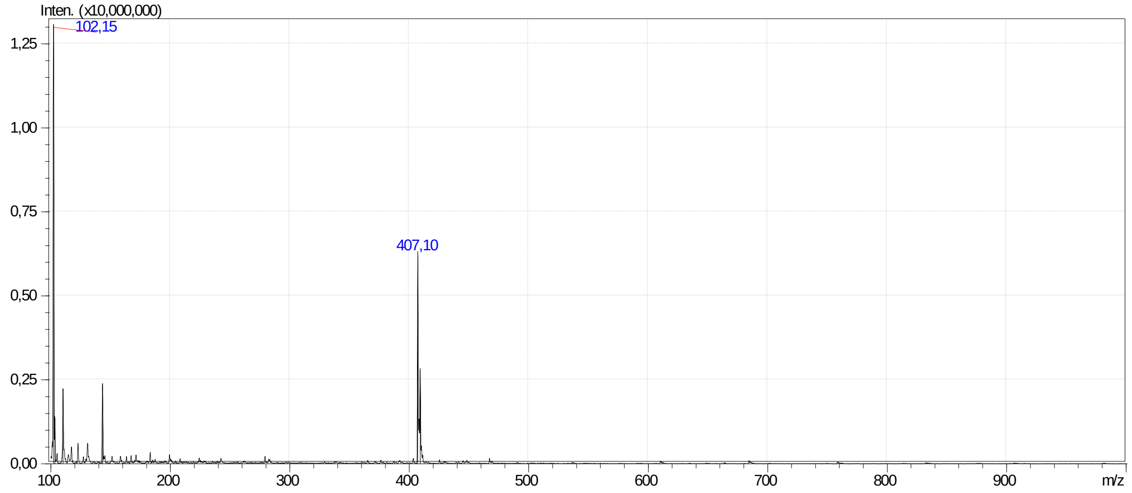

[MS Spectrum]

# of Peaks 6

Raw Spectrum [0,034->0,541],(scan:[3->33])

Background No Background Spectrum

Base Peak m/z 102,15 (Inten : 12.997.389)

m/z	Absolute Intensity	Relative Intensity
102,15	12997389	100,00
103,25	1374497	10,58
110,10	2234970	17,20
143,20	2391259	18,40
407,10	6323564	48,65

Spectra 18. LCMSMS spectra of compound **3e**

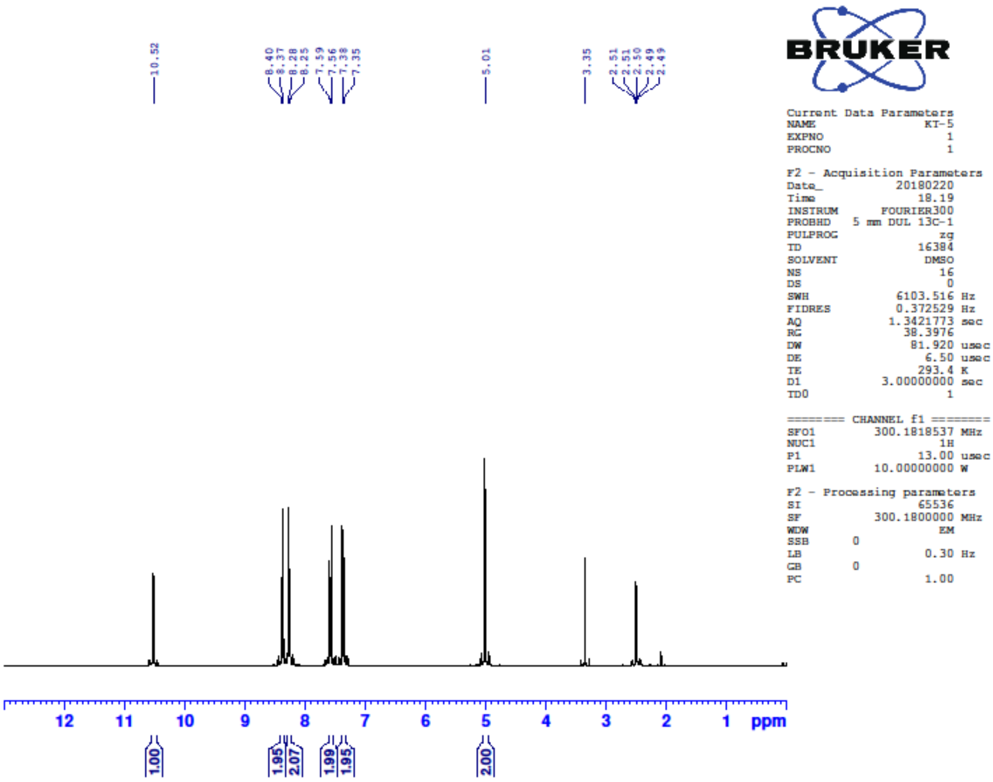

Spectra 19. ^{1}H-NMR spectra of compound **3e**

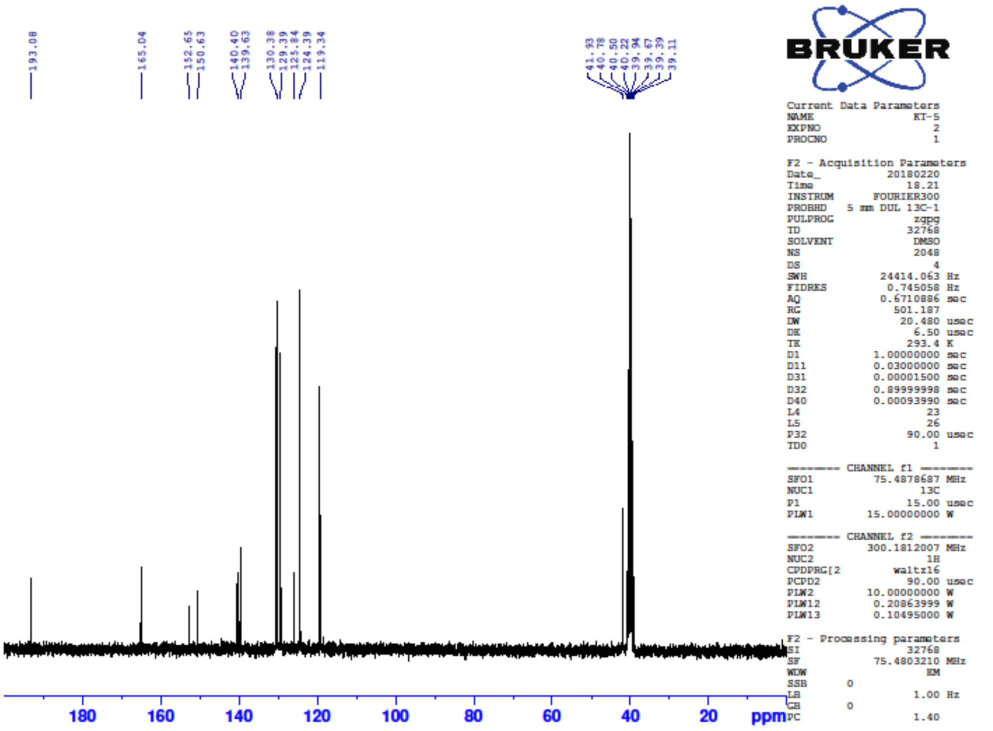

Spectra 20. ^{13}C-NMR spectra of compound **3e**

DOPNALAB

	Item	Value
	Acquired Date&Time	2.07.2018 10:42:41
	Acquired by	System Administrator
	Filename	C:\Users\dopnalab\Desktop\derya\kt seris\kt-61.ispd
	Spectrum name	kt-61
	Sample name	KT-6
	Sample ID	
	Option	
	Comment	
	No. of Scans	10
	Resolution	4 [cm-1]
	Apodization	Happ-Genzel

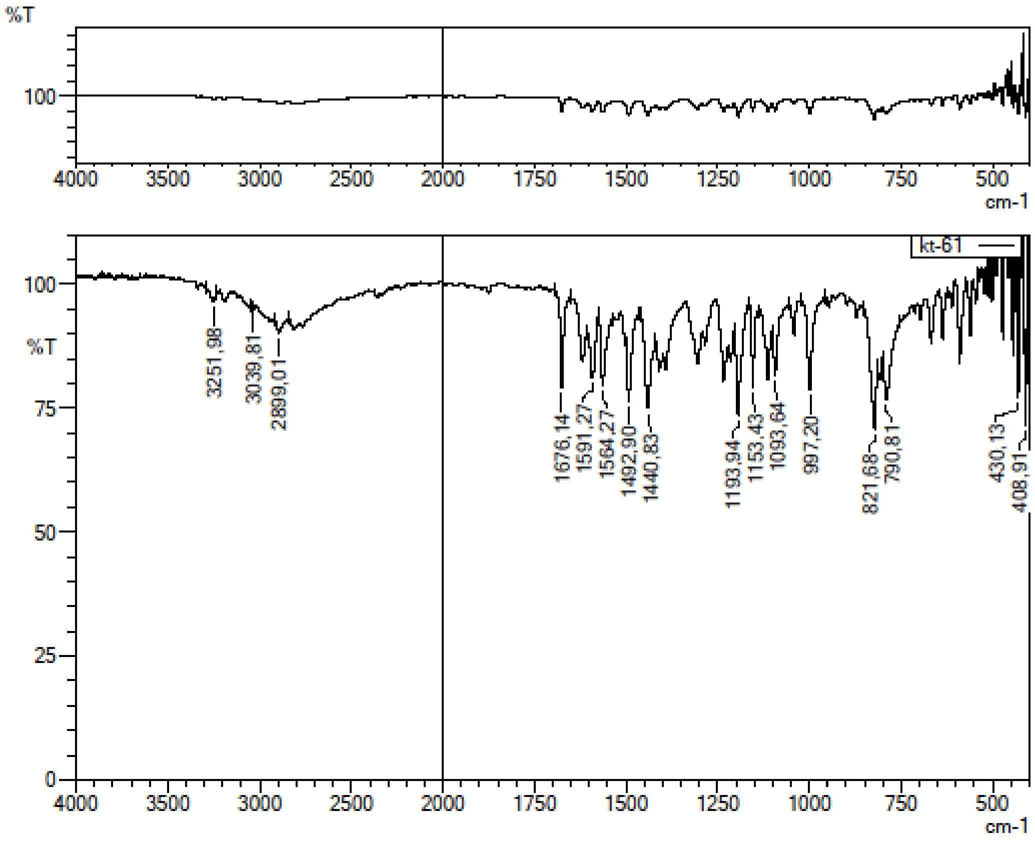

Spectra 21. IR spectra of compound **3f**

LCMSMS ANALYSES REPORT

Sample Name	:KT-6		
Sample ID	:		
Data Filename	: KT-6_derya _029.lcd		
Method Filename	: genel.lcm		
Batch Filename	: batch.lcb		
Vial #	: 1-51		
Injection Volume	: 0,3 uL	Sample Type	: Unknown
Date Acquired	: 22.11.2017 20:38:50	Acquired by	: System Administrator
Date Processed	: 22.11.2017 20:40:51	Processed by	: System Administrator

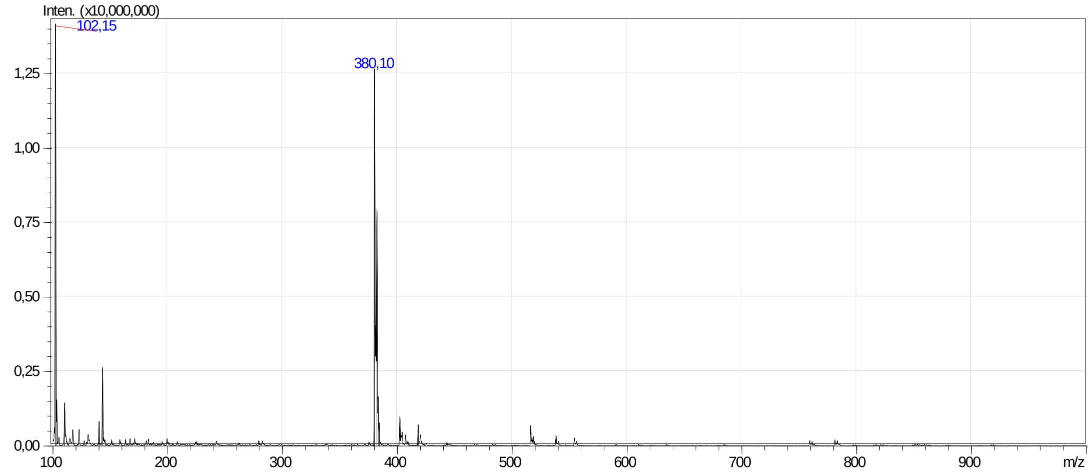

[MS Spectrum]

# of Peaks 11

Raw Spectrum [0,034->0,474],(scan:[3->29])

Background No Background Spectrum

Base Peak m/z 102,15 (Inten : 14.107.810)

m/z	Absolute Intensity	Relative Intensity
102,15	14107810	100,00
103,25	1513541	10,73
110,15	1443692	10,23
140,10	831155	5,89
143,20	2643508	18,74
380,10	12666716	89,79
382,15	7892870	55,95

Spectra 22. LCMSMS spectra of compound **3f**

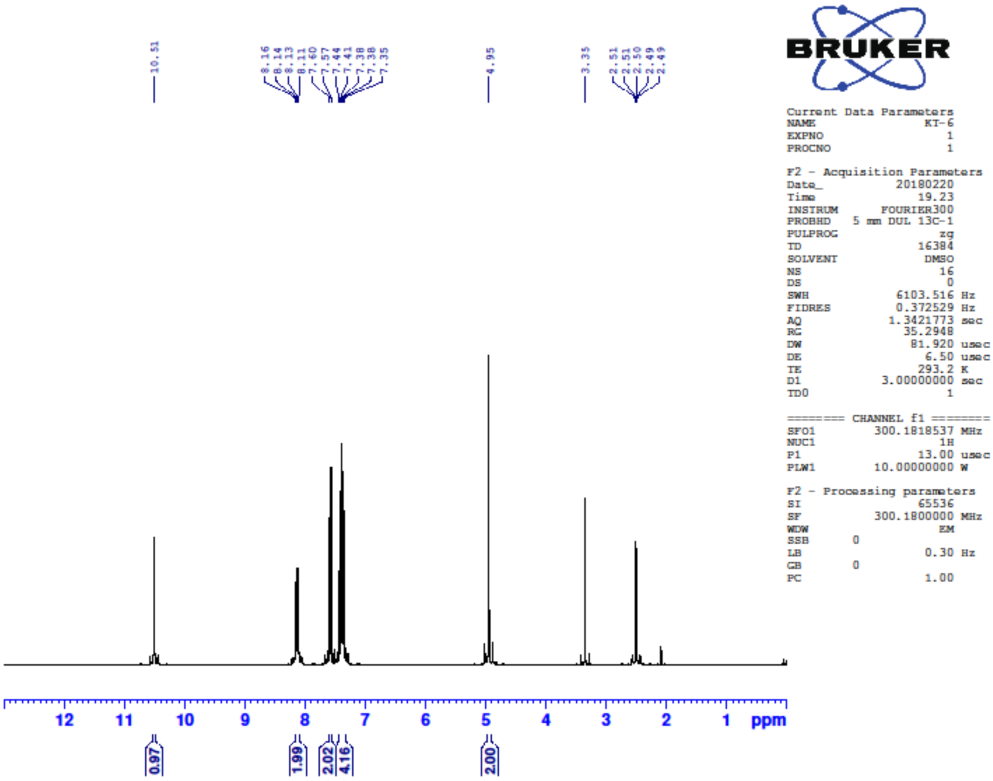

Spectra 23. ^{1}H-NMR spectra of compound **3f**

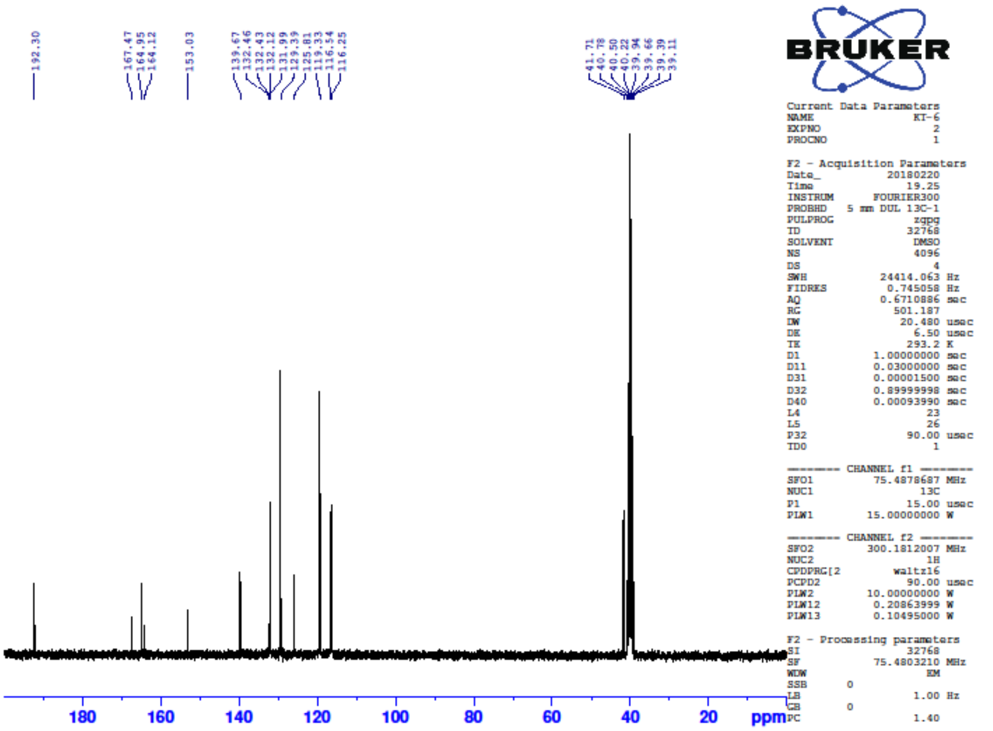

Spectra 24. ^{13}C-NMR spectra of compound **3f**

DOPNALAB

	Item	Value
	Acquired Date&Time	2.07.2018 10:45:43
	Acquired by	System Administrator
	Filename	C:\Users\dopnalab\Desktop\derya\kt seris\kt-71.ispd
	Spectrum name	kt-71
	Sample name	KT-7
	Sample ID	
	Option	
	Comment	
	No. of Scans	10
	Resolution	4 [cm-1]
	Apodization	Happ-Genzel

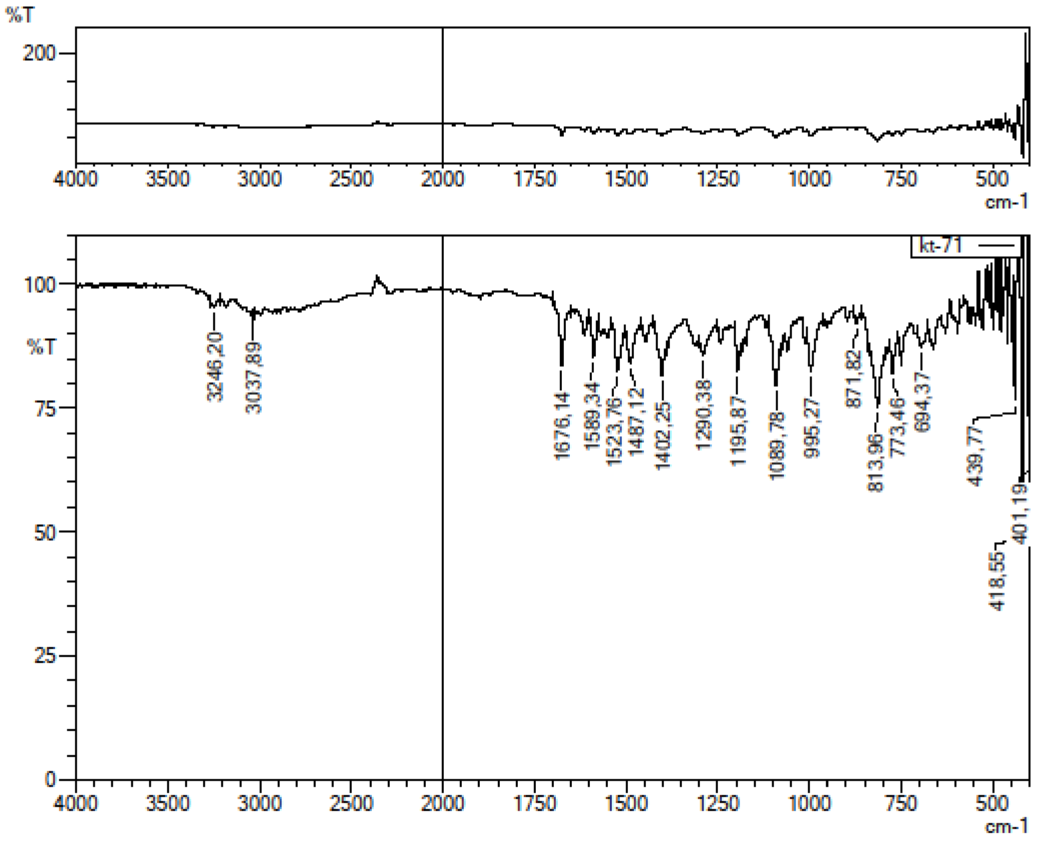

Spectra 25. IR spectra of compound **3g**

LCMSMS ANALYSES REPORT

Sample Name	:KT-7		
Sample ID	:		
Data Filename	: KT-7_derya _030.lcd		
Method Filename	: genel.lcm		
Batch Filename	: batch.lcb		
Vial #	: 1-52		
Injection Volume	: 0,3 uL	Sample Type	: Unknown
Date Acquired	: 22.11.2017 20:41:28	Acquired by	: System Administrator
Date Processed	: 22.11.2017 20:43:30	Processed by	: System Administrator

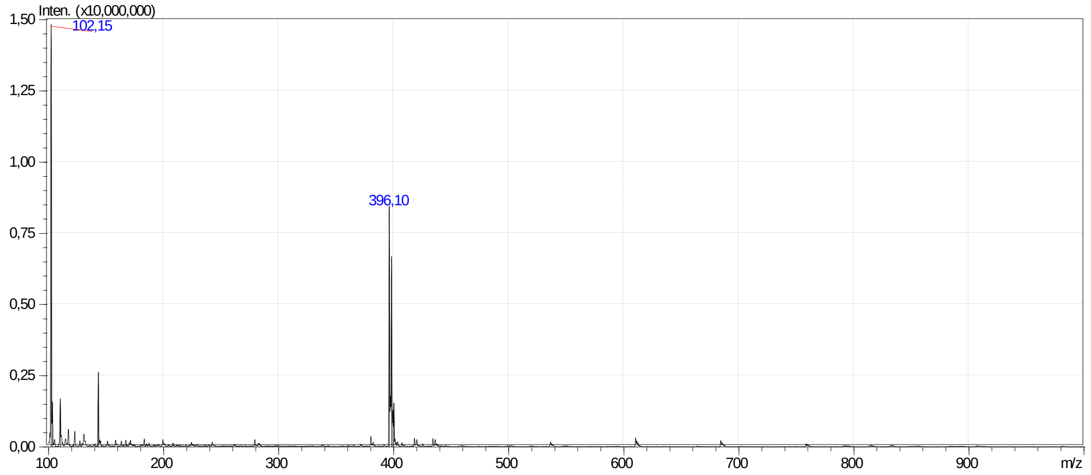

[MS Spectrum]

# of Peaks 8

Raw Spectrum [0,034->0,440],(scan:[3->27])

Background No Background Spectrum

Base Peak m/z 102,15 (Inten : 14.770.606)

m/z	Absolute Intensity	Relative Intensity
102,15	14770606	100,00
103,25	1548008	10,48
110,10	1702875	11,53
143,20	2619854	17,74
396,10	8463067	57,30
398,05	6622719	44,84
399,10	1283522	8,69

Spectra 26. LCMSMS spectra of compound **3g**

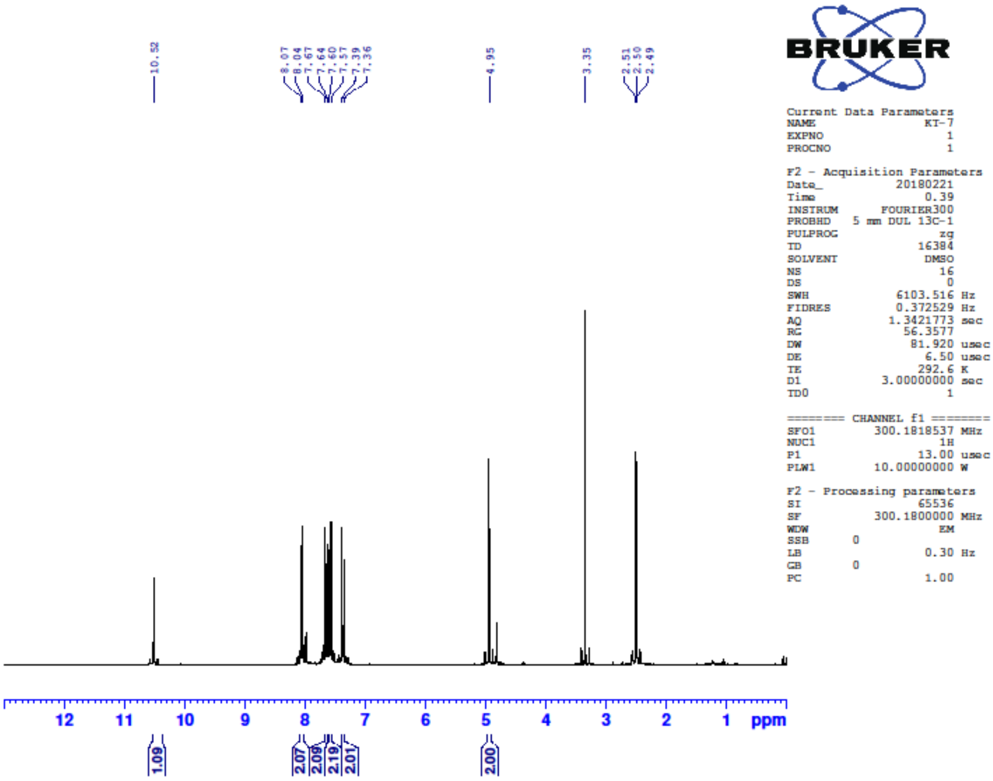

Spectra 27. ^{1}H-NMR spectra of compound **3g**

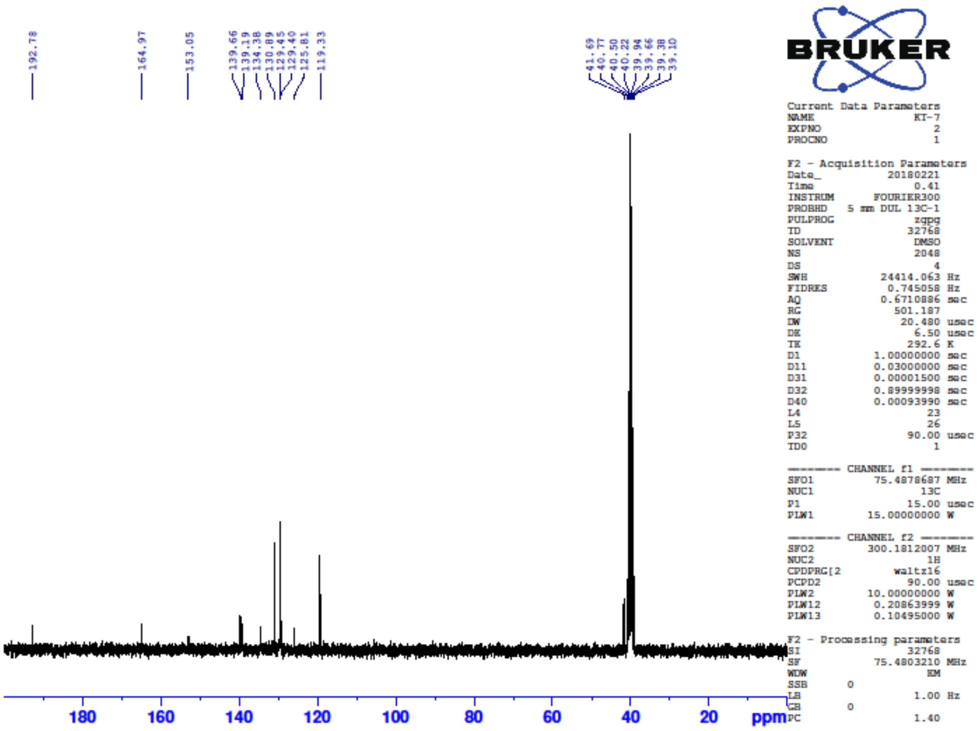

Spectra 28. ^{13}C-NMR spectra of compound **3g**

DOPNALAB

Item	Value
Acquired Date&Time	2.07.2018 10:49:45
Acquired by	System Administrator
Filename	C:\Users\dopnalab\Desktop\derya\kt seris\kt-81.ispd
Spectrum name	kt-81
Sample name	KT-8
Sample ID	
Option	
Comment	
No. of Scans	10
Resolution	4 [cm-1]
Apodization	Happ-Genzel

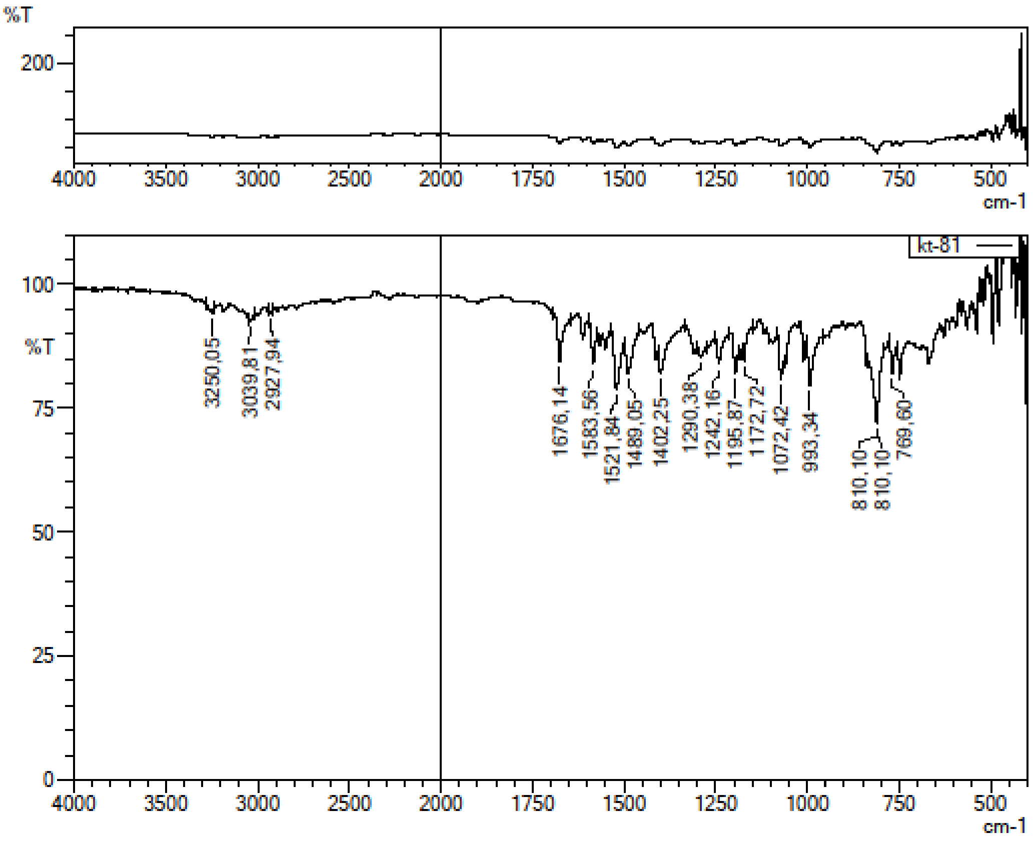

Spectra 29. IR spectra of compound **3h**

LCMSMS ANALYSES REPORT

Sample Name	:KT-8		
Sample ID	:		
Data Filename	: KT-8_derya _031.lcd		
Method Filename	: genel.lcm		
Batch Filename	: batch.lcb		
Vial #	: 1-53		
Injection Volume	: 0,3 uL	Sample Type	: Unknown
Date Acquired	: 22.11.2017 20:44:05	Acquired by	: System Administrator
Date Processed	: 22.11.2017 20:46:06	Processed by	: System Administrator

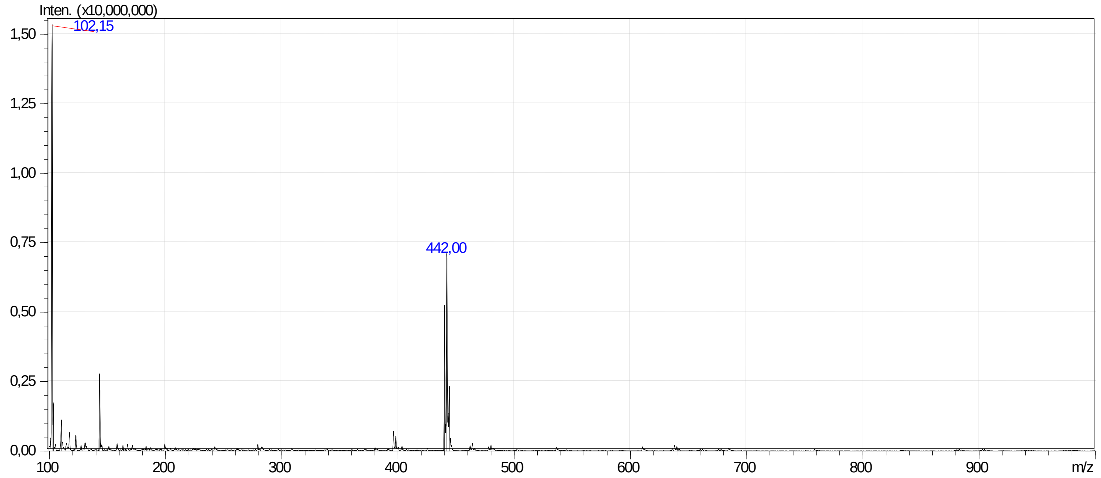

[MS Spectrum]

# of Peaks 7

Raw Spectrum [0,034->0,406],(scan:[3->25])

Background No Background Spectrum

Base Peak m/z 102,15 (Inten : 15.311.210)

m/z	Absolute Intensity	Relative Intensity
102,15	15311210	100,00
103,25	1697449	11,09
110,15	1117373	7,30
143,20	2783947	18,18
440,05	5205925	34,00
442,00	7109232	46,43

Spectra 30. LCMSMS spectra of compound **3h**

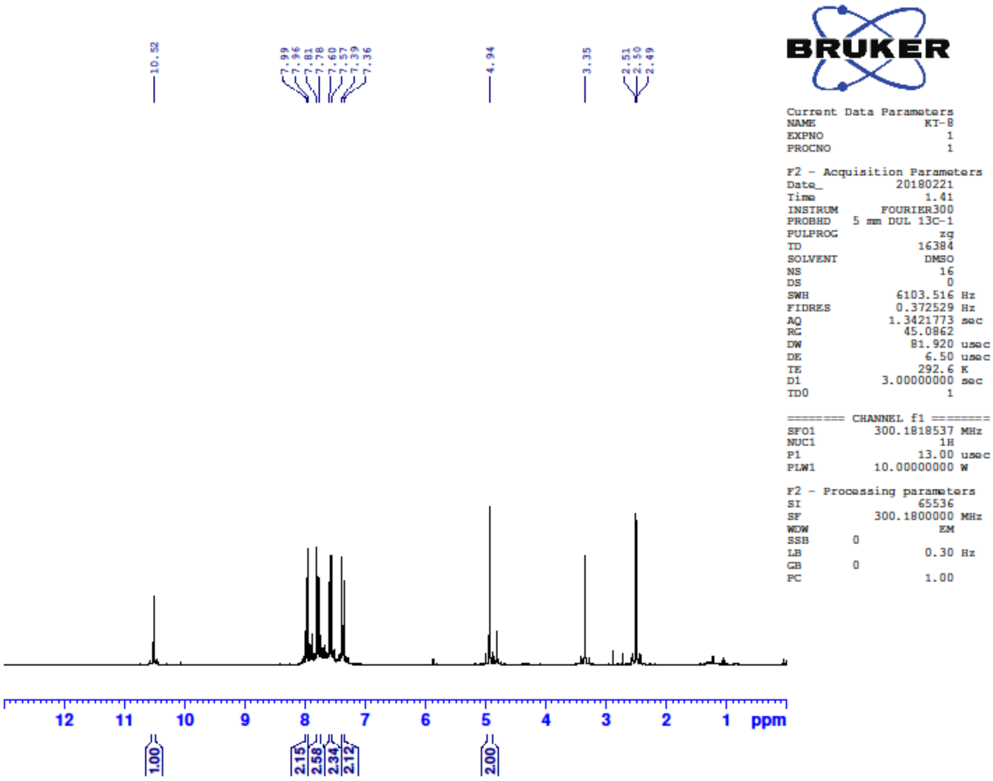

Spectra 31. ^{1}H-NMR spectra of compound **3h**

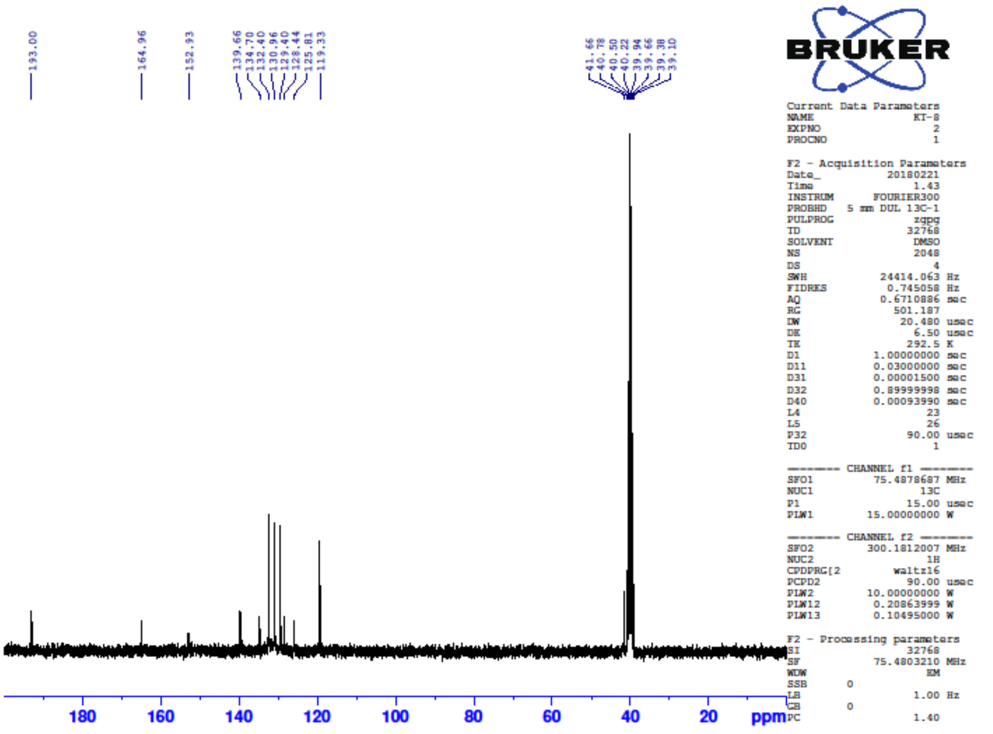

Spectra 32. ^{13}C-NMR spectra of compound **3h**

DOPNALAB

Item	Value
Acquired Date&Time	2.07.2018 10:52:07
Acquired by	System Administrator
Filename	C:\Users\dopnalab\Desktop\derya\kt seris\kt-91.ispd
Spectrum name	kt-91
Sample name	KT-9
Sample ID	
Option	
Comment	
No. of Scans	10
Resolution	4 [cm-1]
Apodization	Happ-Genzel

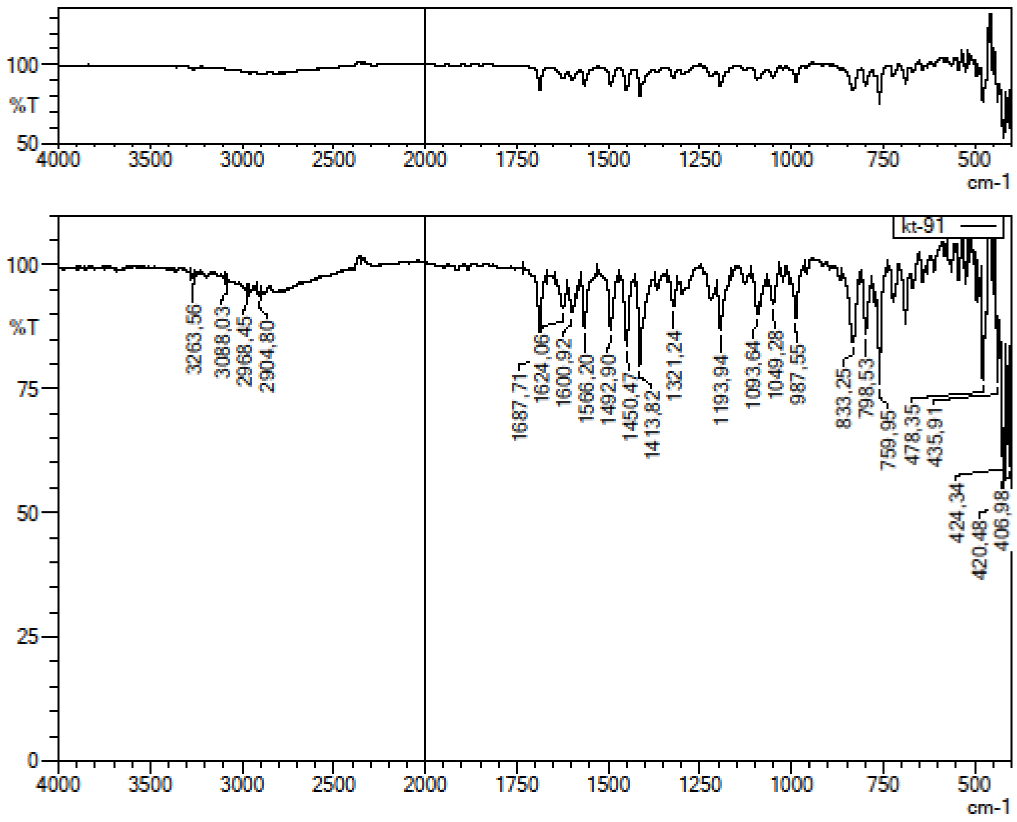

Spectra 33. IR spectra of compound **3i**

LCMSMS ANALYSES REPORT

Sample Name	:KT-9		
Sample ID	:		
Data Filename	: KT-9_derya _032.lcd		
Method Filename	: genel.lcm		
Batch Filename	: batch.lcb		
Vial #	: 1-54		
Injection Volume	: 0,3 uL	Sample Type	: Unknown
Date Acquired	: 22.11.2017 20:46:41	Acquired by	: System Administrator
Date Processed	: 22.11.2017 20:48:43	Processed by	: System Administrator

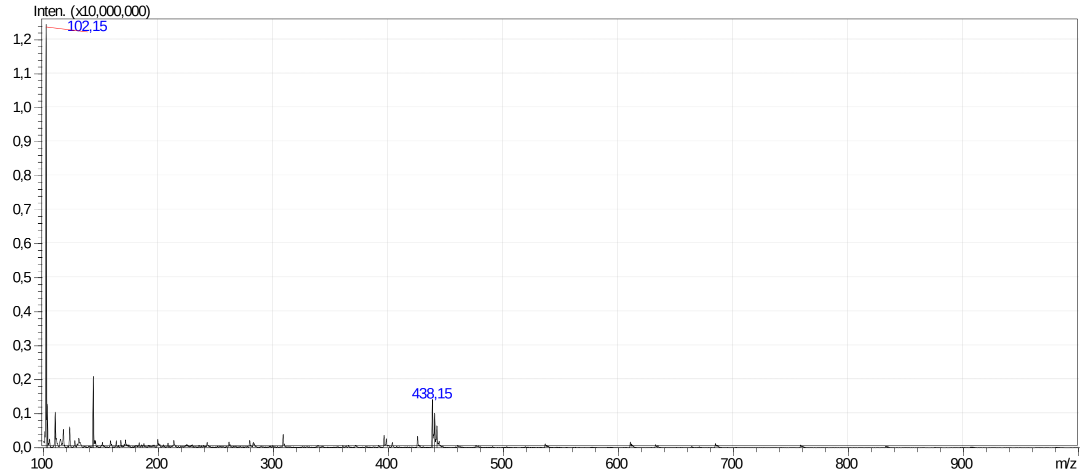

[MS Spectrum]

# of Peaks	7
Raw Spectrum	[0,034->0,541],(scan:[3->33])
Background	No Background Spectrum
Base Peak	m/z 102,15 (Inten : 12.377.799)

m/z	Absolute Intensity	Relative Intensity
102,15	12377799	100,00
103,20	1285564	10,39
110,10	1053057	8,51
143,20	2099402	16,96
438,15	1417140	11,45

Spectra 34. LCMSMS spectra of compound **3i**

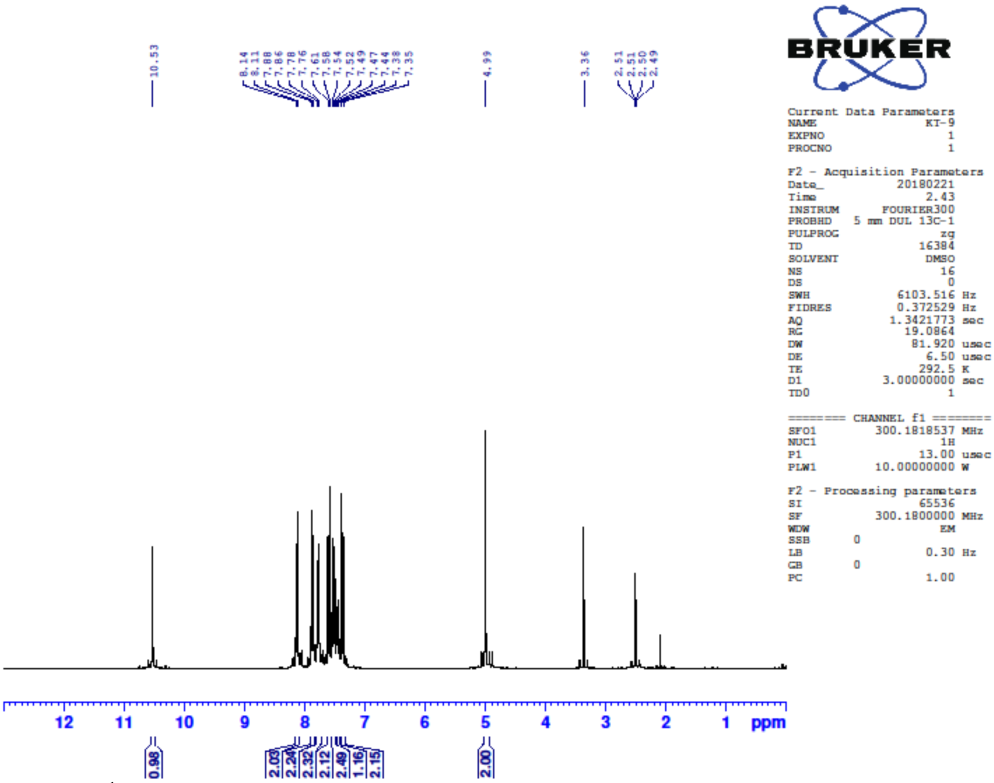

Spectra 35. ^{1}H-NMR spectra of compound **3i**

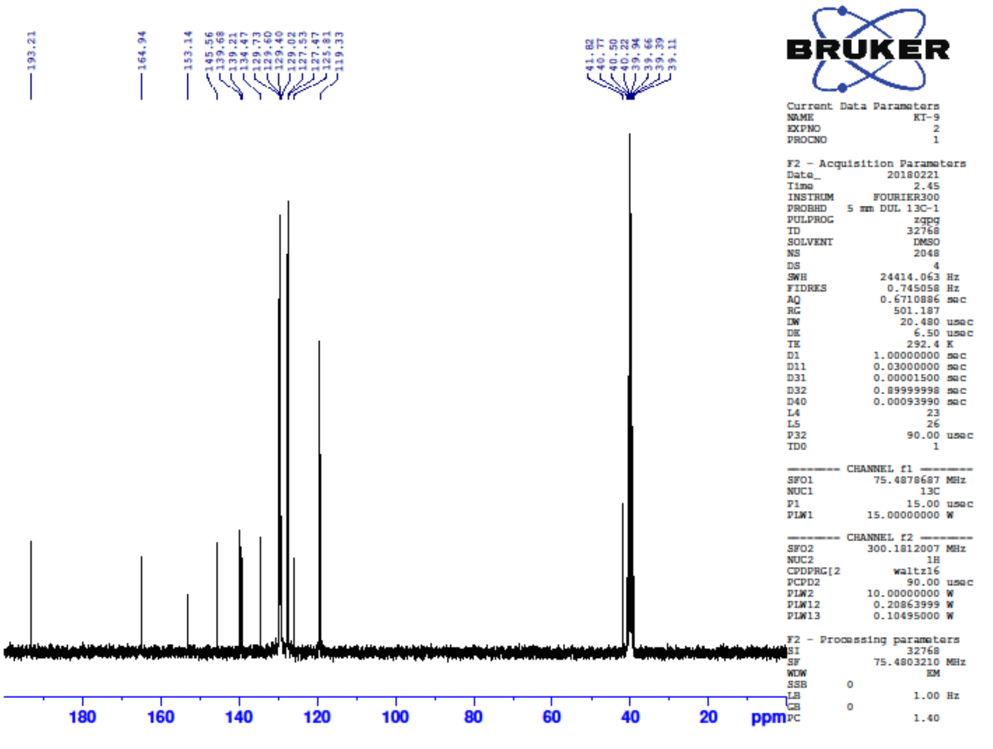

Spectra 36. ^{13}C-NMR spectra of compound **3i**

DOPNALAB

	Item	Value
	Acquired Date&Time	2.07.2018 10:56:09
	Acquired by	System Administrator
	Filename	C:\Users\dopnalab\Desktop\derya\kt seris\kt-102.ispd
	Spectrum name	kt-102
	Sample name	KT-10
	Sample ID	
	Option	
	Comment	
	No. of Scans	10
	Resolution	4 [cm-1]
	Apodization	Happ-Genzel

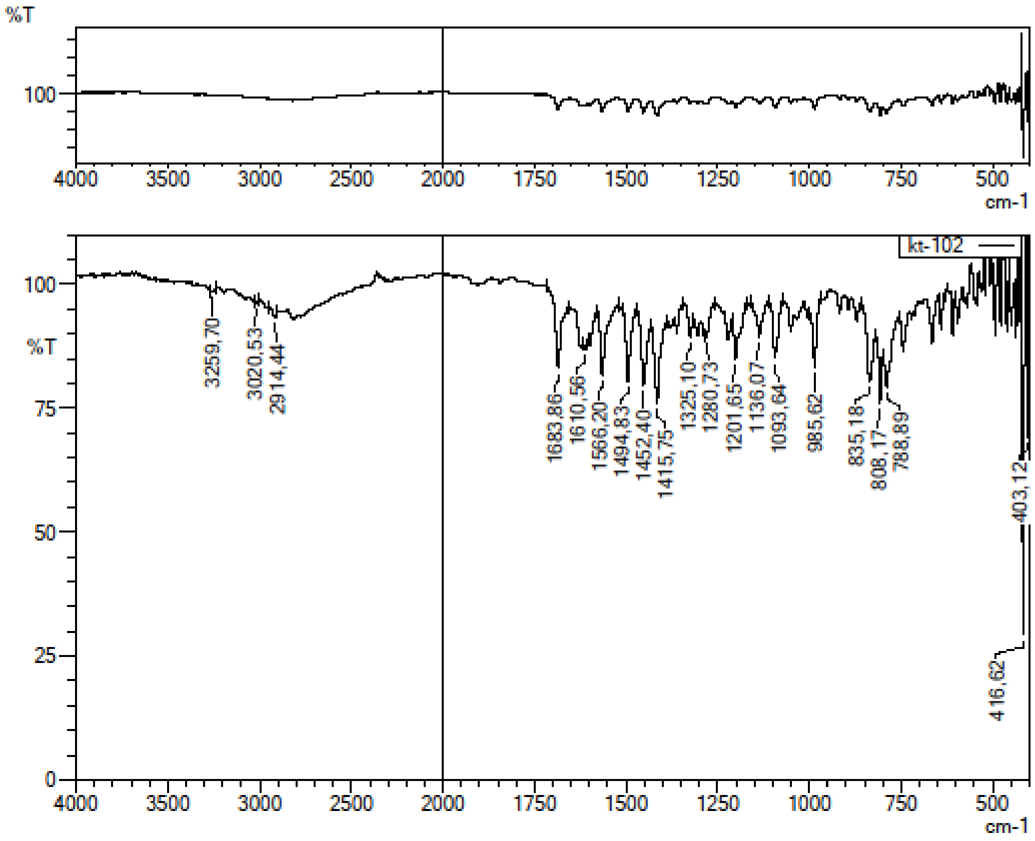

Spectra 37. IR spectra of compound **3j**

LCMSMS ANALYSES REPORT

Sample Name	:KT-10		
Sample ID	:		
Data Filename	: KT-10_derya _033.lcd		
Method Filename	: genel.lcm		
Batch Filename	: batch.lcb		
Vial #	: 1-55		
Injection Volume	: 0,3 uL	Sample Type	: Unknown
Date Acquired	: 22.11.2017 20:49:18	Acquired by	: System Administrator
Date Processed	: 22.11.2017 20:21:50	Processed by	: System Administrator

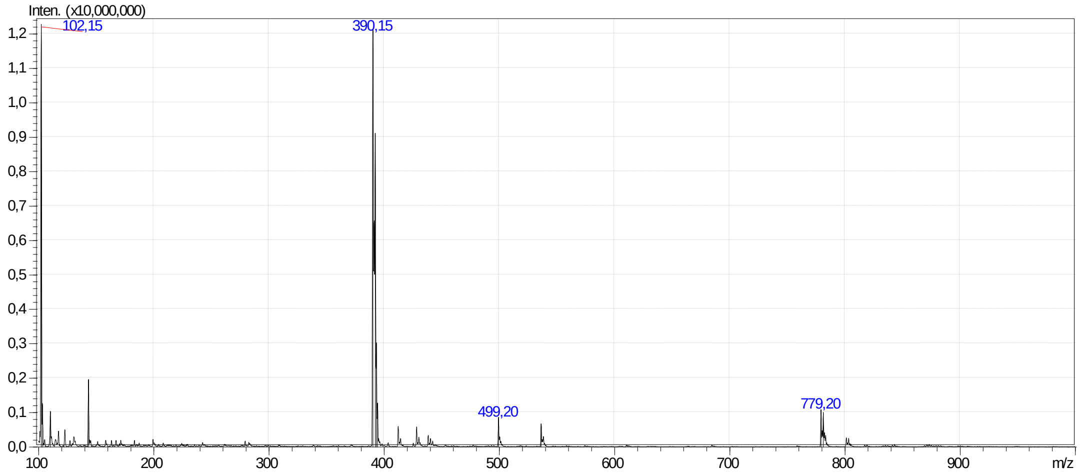

[MS Spectrum]

# of Peaks 12

Raw Spectrum [0,000->0,507],(scan:[1->31])

Background No Background Spectrum

Base Peak m/z 102,15 (Inten : 12.207.922)

m/z	Absolute Intensity	Relative Intensity
102,15	12207922	100,00
103,25	1229997	10,08
110,10	1041175	8,53
143,20	1963218	16,08
390,15	12126320	99,33
392,15	9117061	74,68

Spectra 38. LCMSMS spectra of compound **3j**

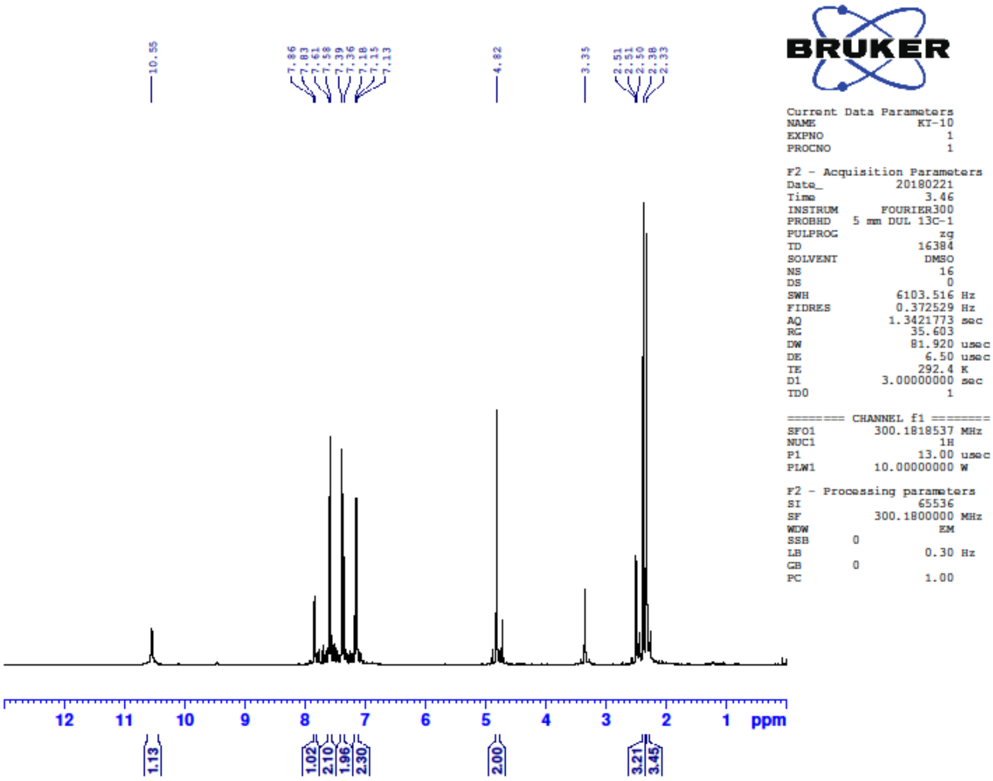

Spectra 39. ^{1}H-NMR spectra of compound **3j**

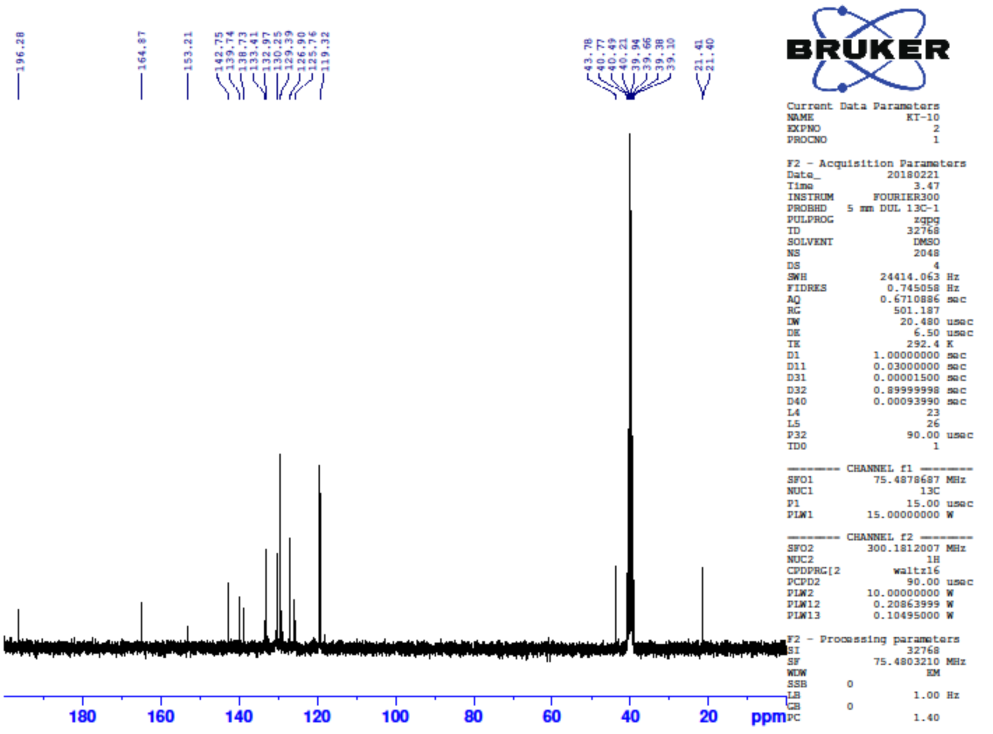

Spectra 40. ^{13}C-NMR spectra of compound **3j**

DOPNALAB

Item	Value
Acquired Date&Time	2.07.2018 11:03:02
Acquired by	System Administrator
Filename	C:\Users\dopnalab\Desktop\derya\kt seris\kt-122.ispd
Spectrum name	kt-122
Sample name	KT-12
Sample ID	
Option	
Comment	
No. of Scans	10
Resolution	4 [cm-1]
Apodization	Happ-Genzel

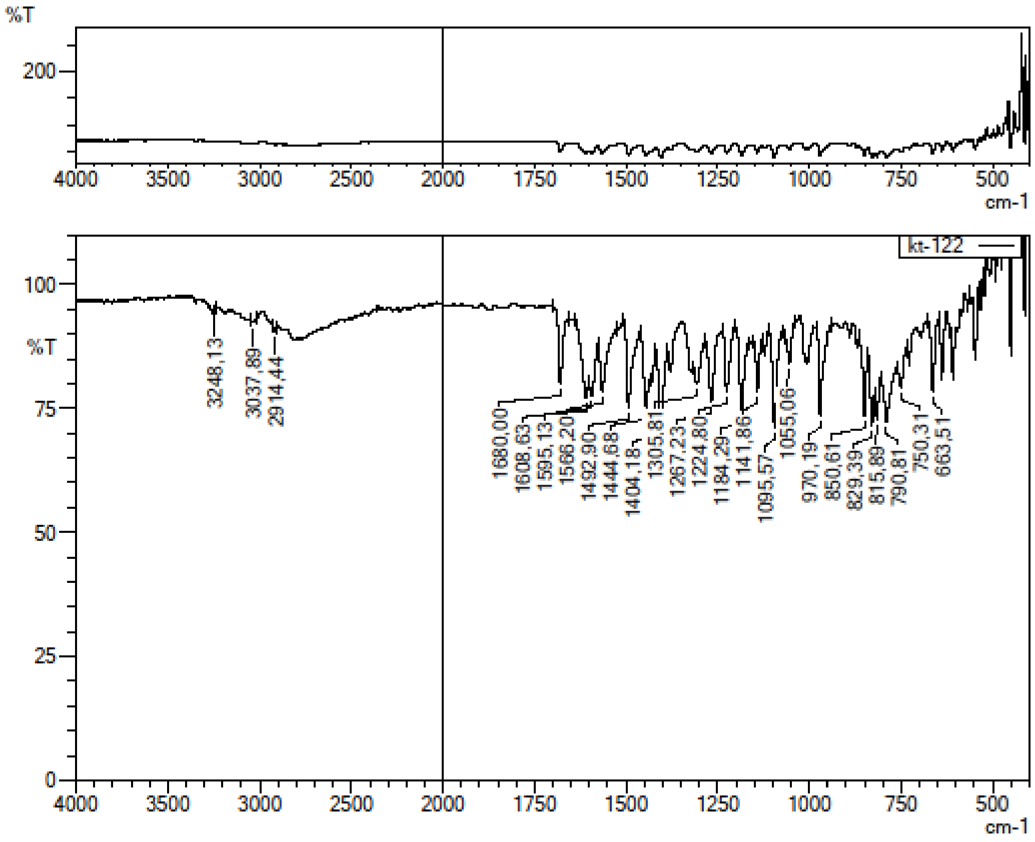

Spectra 41. IR spectra of compound **3k**

LCMSMS ANALYSES REPORT

Sample Name	:KT-12		
Sample ID	:		
Data Filename	: KT-12_derya _035.lcd		
Method Filename	: genel.lcm		
Batch Filename	: batch.lcb		
Vial #	: 1-57		
Injection Volume	: 0,3 uL	Sample Type	: Unknown
Date Acquired	: 22.11.2017 20:54:38	Acquired by	: System Administrator
Date Processed	: 22.11.2017 20:56:40	Processed by	: System Administrator

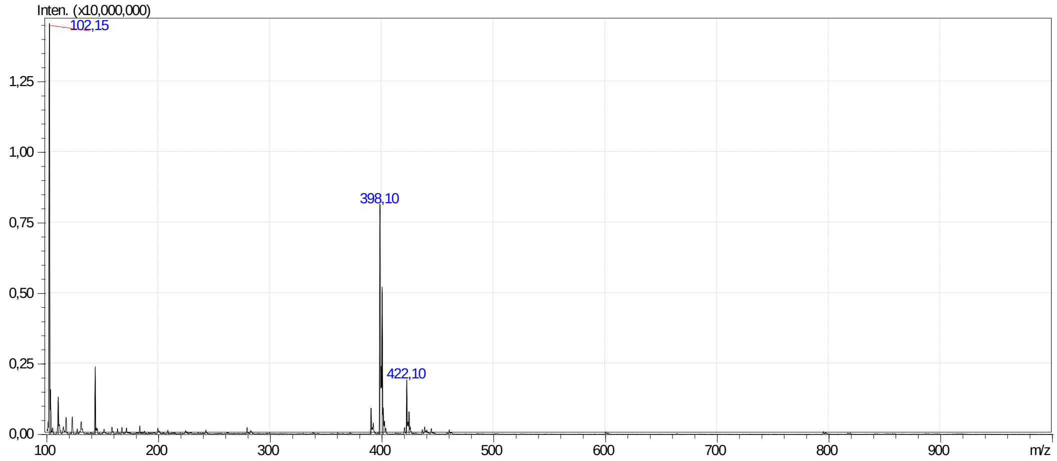

[MS Spectrum]

# of Peaks 10

Raw Spectrum [0,034->0,440],(scan:[3->27])

Background No Background Spectrum

Base Peak m/z 102,15 (Inten : 14.501.980)

m/z	Absolute Intensity	Relative Intensity
102,15	14501980	100,00
103,25	1552690	10,71
110,10	1338285	9,23
143,20	2405257	16,59
390,15	929008	6,41
398,10	8166225	56,31
400,10	5228954	36,06

Spectra 42. LCMSMS spectra of compound **3k**

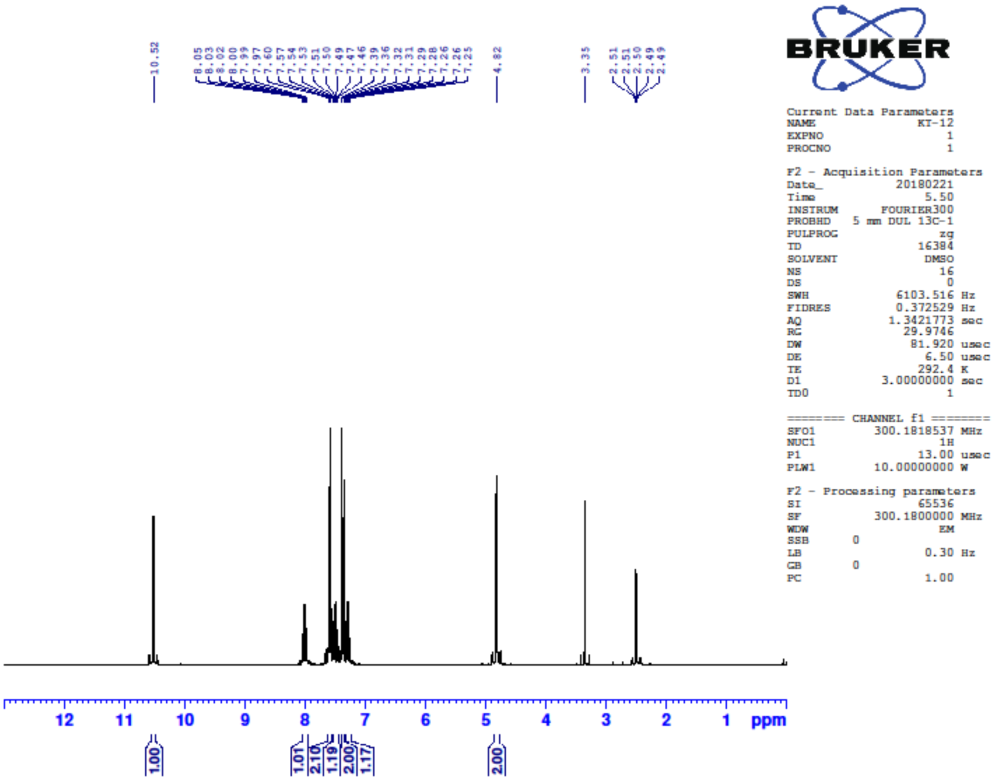

Spectra 43. ^{1}H-NMR spectra of compound **3k**

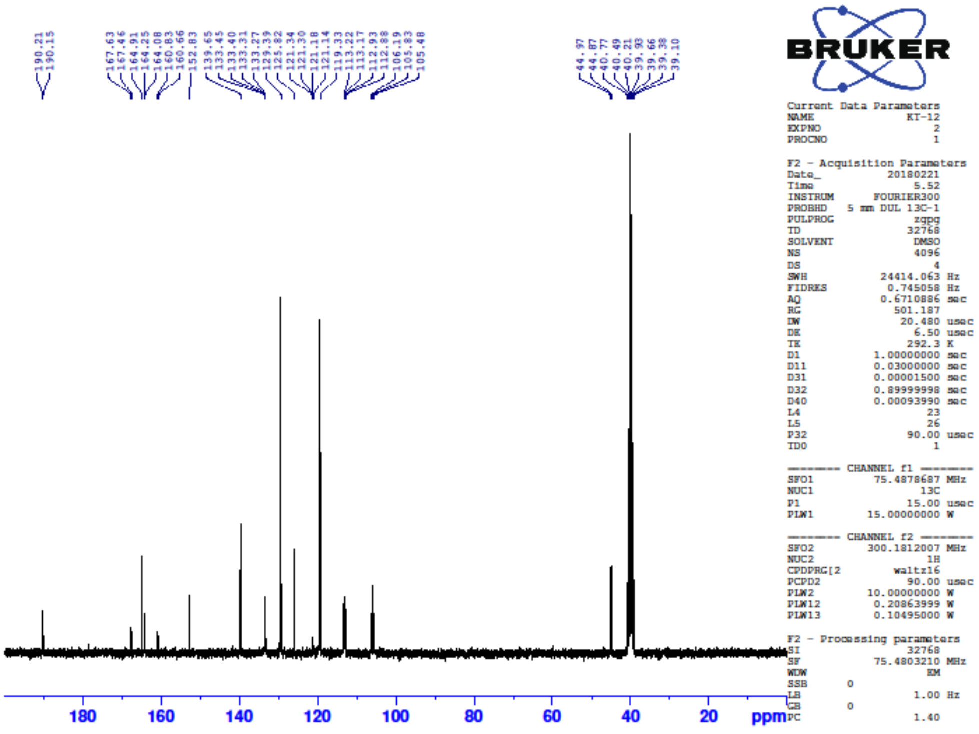

Spectra 44. ^{13}C-NMR spectra of compound **3k**

DOPNALAB

Item	Value
Acquired Date&Time	2.07.2018 11:05:16
Acquired by	System Administrator
Filename	C:\Users\dopnalab\Desktop\derya\kt seris\kt-131.ispd
Spectrum name	kt-131
Sample name	KT-13
Sample ID	
Option	
Comment	
No. of Scans	10
Resolution	4 [cm-1]
Apodization	Happ-Genzel

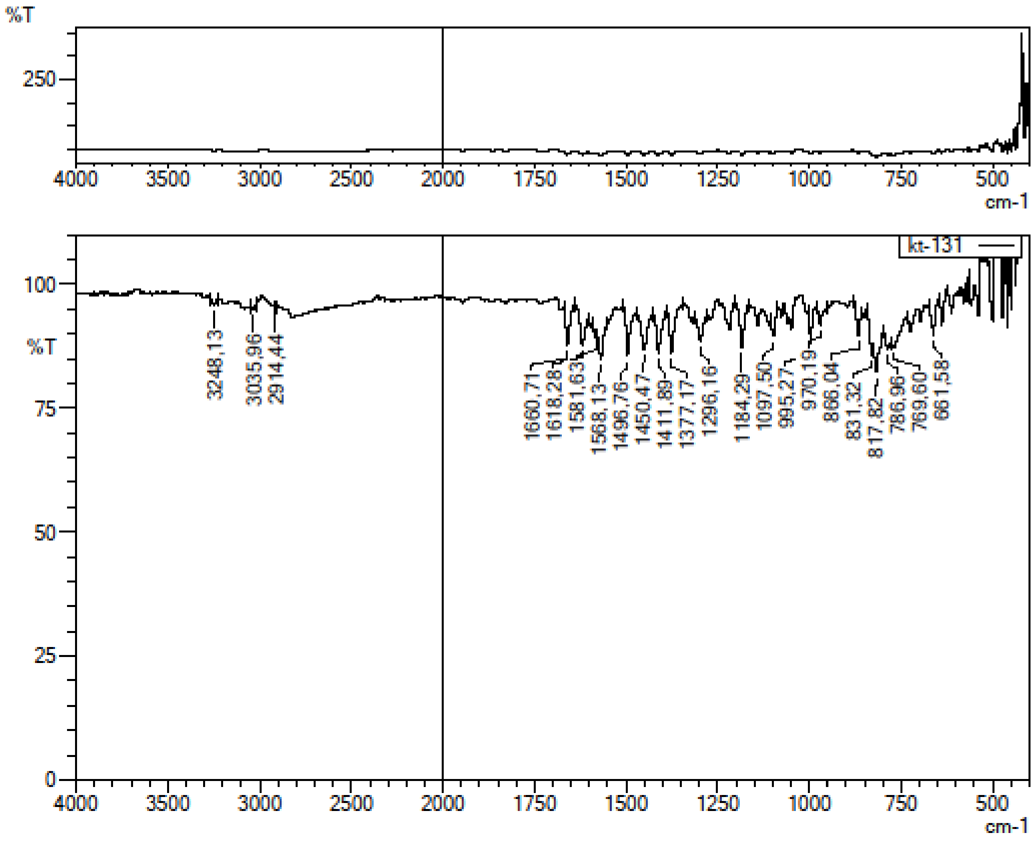

Spectra 45. IR spectra of compound **3l**

LCMSMS ANALYSES REPORT

Sample Name	:KT-13		
Sample ID	:		
Data Filename	: KT-13_derya _036.lcd		
Method Filename	: genel.lcm		
Batch Filename	: batch.lcb		
Vial #	: 1-58		
Injection Volume	: 0,3 uL	Sample Type	: Unknown
Date Acquired	: 22.11.2017 20:57:18	Acquired by	: System Administrator
Date Processed	: 22.11.2017 20:59:24	Processed by	: System Administrator

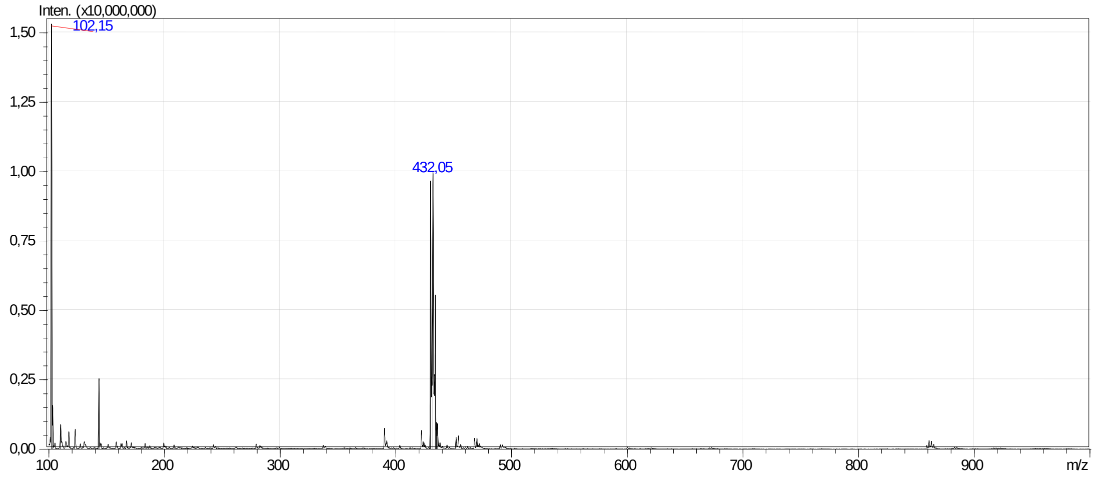

[MS Spectrum]

# of Peaks	9
Raw Spectrum	[0,034->0,406],(scan:[3->25])
Background	No Background Spectrum
Base Peak	m/z 102,15 (Inten : 15.251.295)

m/z	Absolute Intensity	Relative Intensity
102,15	15251295	100,00
103,25	1534719	10,06
110,10	892258	5,85
143,20	2541071	16,66
430,00	9671580	63,41
432,05	9958278	65,29
434,05	5519242	36,19

Spectra 46. LCMSMS spectra of compound **3l**

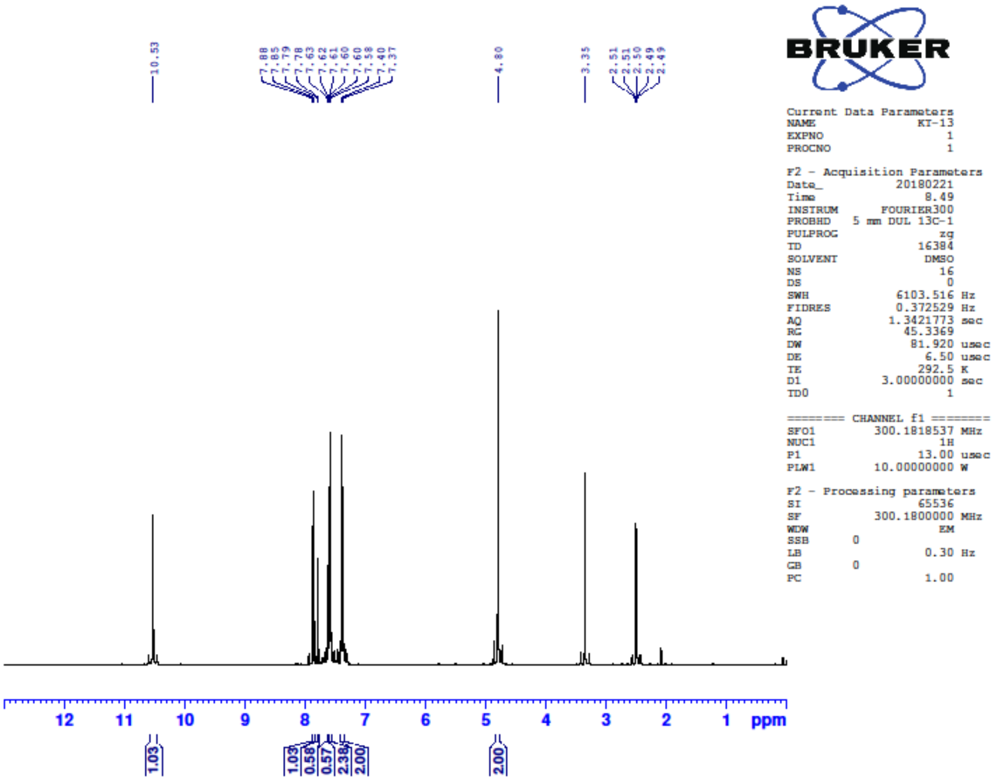

Spectra 47. ^{1}H-NMR spectra of compound **3l**

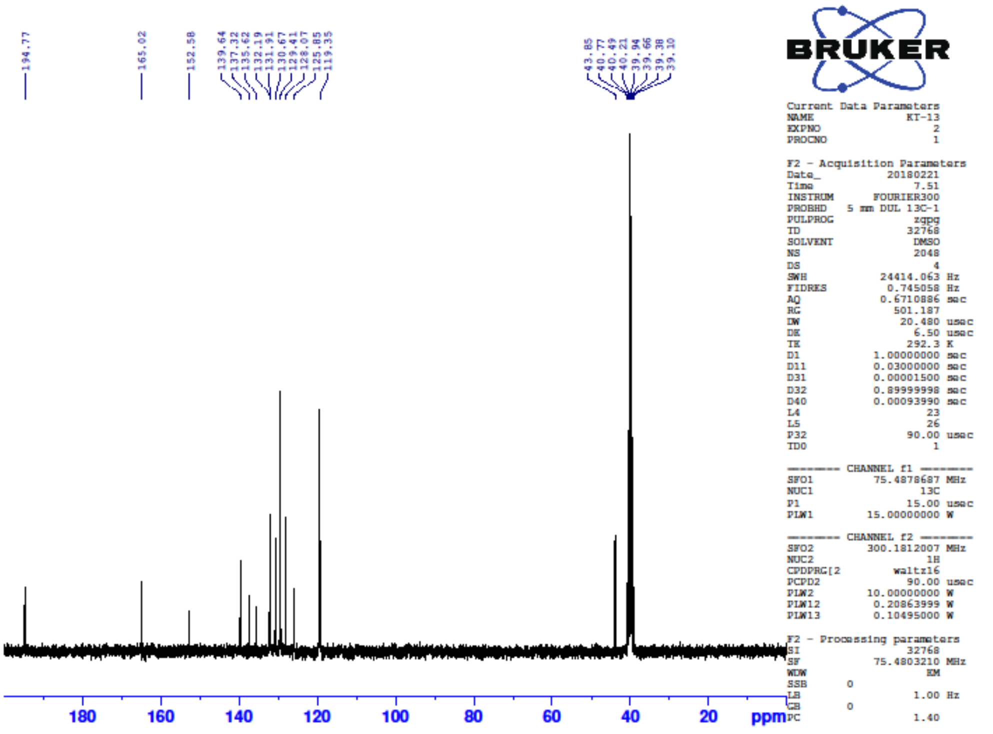

Spectra 48. ^{13}C-NMR spectra of compound **3l**
